# Integrative oncogene-dependency mapping identifies RIT1 vulnerabilities and synergies in lung cancer

**DOI:** 10.1038/s41467-021-24841-y

**Published:** 2021-08-09

**Authors:** Athea Vichas, Amanda K. Riley, Naomi T. Nkinsi, Shriya Kamlapurkar, Phoebe C. R. Parrish, April Lo, Fujiko Duke, Jennifer Chen, Iris Fung, Jacqueline Watson, Matthew Rees, Austin M. Gabel, James D. Thomas, Robert K. Bradley, John K. Lee, Emily M. Hatch, Marina K. Baine, Natasha Rekhtman, Marc Ladanyi, Federica Piccioni, Alice H. Berger

**Affiliations:** 1 Human Biology Division, Fred Hutchinson Cancer Research Center, Seattle, WA USA; 2 Molecular and Cellular Biology Program, University of Washington, Seattle, WA USA; 3 Department of Genome Sciences, University of Washington, Seattle, WA USA; 4 Broad Institute of MIT & Harvard, Cambridge, MA USA; 5 Medical Scientist Training Program, University of Washington, Seattle, WA USA; 6 Computational Biology Program, Public Health Sciences Division, Fred Hutchinson Cancer Research Center, Seattle, WA USA; 7 Basic Sciences Division, Fred Hutchinson Cancer Research Center, Seattle, WA USA; 8 Department of Pathology, Memorial Sloan Kettering Cancer Center, New York, USA; 9 Present Address: Merck Research Laboratories, Boston, MA USA

**Keywords:** High-throughput screening, Targeted therapies, Non-small-cell lung cancer

## Abstract

CRISPR-based cancer dependency maps are accelerating advances in cancer precision medicine, but adequate functional maps are limited to the most common oncogenes. To identify opportunities for therapeutic intervention in other rarer subsets of cancer, we investigate the oncogene-specific dependencies conferred by the lung cancer oncogene, *RIT1*. Here, genome-wide CRISPR screening in *KRAS*, *EGFR*, and *RIT1*-mutant isogenic lung cancer cells identifies shared and unique vulnerabilities of each oncogene. Combining this genetic data with small-molecule sensitivity profiling, we identify a unique vulnerability of *RIT1*-mutant cells to loss of spindle assembly checkpoint regulators. Oncogenic RIT1^M90I^ weakens the spindle assembly checkpoint and perturbs mitotic timing, resulting in sensitivity to Aurora A inhibition. In addition, we observe synergy between mutant *RIT1* and activation of YAP1 in multiple models and frequent nuclear overexpression of YAP1 in human primary *RIT1*-mutant lung tumors. These results provide a genome-wide atlas of oncogenic *RIT1* functional interactions and identify components of the RAS pathway, spindle assembly checkpoint, and Hippo/YAP1 network as candidate therapeutic targets in *RIT1*-mutant lung cancer.

## Abstract (web summary)

*RIT1* mutations are mutually exclusive with other lung cancer drivers and lack targeted therapies. Here the authors examine genetic dependencies of mutant *RIT1* with genome-wide CRISPR screens, revealing synergy between RIT1 and YAP1, and increased sensitivity to Aurora kinase inhibitors.

## Introduction

Somatic mutations that activate EGFR/RAS pathway signaling are a hallmark of lung adenocarcinoma, occurring in more than 75% of tumors^1^. Oncogenes in the EGFR/RAS pathway display ‘oncogene addiction’, a tumor-specific reliance on sustained cell signaling for cell survival, and consequently these mutated oncogenes represent powerful drug targets for lung cancer therapy^2^. Several of the mutated genes in this pathway are clinically targeted to improve outcomes for lung cancer patients. For example, somatic mutations in *EGFR* underlie sensitivity to EGFR inhibitors erlotinib and osimertinib^3,4^, and chromosomal rearrangements involving *ALK* underlie sensitivity to crizotinib^5^ and other ALK inhibitors.

Recently, we connected RIT1 to the EGFR/RAS pro-tumorigenic signaling network^6–8^. *RIT1* is mutated in 2% and amplified in 14% of lung adenocarcinomas^6,7^. *RIT1* encodes a ubiquitously expressed RAS-family GTPase protein^9,10^. Mutations in *RIT1* are mutually exclusive with mutations in *KRAS*, *EGFR*, *ALK*, *MET*, and other driver oncogenes in lung adenocarcinoma, suggesting that *RIT1* may drive EGFR/RAS pathway activation in *RIT1*-mutant tumors^6^. Consistent with this idea, mutant *RIT1* can transform NIH3T3 fibroblasts and confer resistance to EGFR inhibition in *EGFR*-mutant lung cancer cells^7,8^. Mutant *RIT1* has been identified in a patient with acquired resistance to ALK inhibition^11^. Somatic *RIT1* mutations also occur in myeloid malignancies^12^, and focal *RIT1* amplifications are observed in uterine carcinosarcoma^13^. Because *RIT1* mutations are mutually exclusive with other driver alterations in lung adenocarcinoma^6^, patients with *RIT1*-mutant lung tumors have limited therapeutic options of standard chemo- and immuno-therapy regimens but no targeted therapies. Beyond cancer, germline mutations in *RIT1* are found in patients with the RAS-opathy Noonan syndrome (NS)^14^.

Both RIT1 protein abundance^15^ and GTP binding^16^ appear to be central to its oncogenic function. *RIT1* mutations disrupt RIT1’s negative regulation by the ubiquitin adaptor protein LZTR1, leading to increased RIT1 protein abundance. Cancer- and NS-associated variants also display decreased GTP hydrolysis, increased nucleotide exchange, or increased GTP binding^15,16^, and the exact contribution of GTP binding versus protein abundance to RIT1’s oncogenic function remains to be determined. In both cancer and NS, a similar spectrum of *RIT1* missense and in-frame insertion/deletion mutations is observed, with the majority occurring in the switch II domain of the protein. Mutation at methionine 90 (M90I) is recurrent, but the spectrum of mutations is relatively diverse and the majority have been shown to confer the same cellular transformation capability^7^ and loss of LZTR1 binding^15^. Beyond these observations, relatively little is known about the specific mechanism of action of RIT1, how it differs from KRAS, and what proteins are critical to induce its oncogenic function. Further understanding the cellular consequences of oncogenic *RIT1* mutations could open up strategies for therapeutic intervention in *RIT1*-mutant cancers and Noonan syndrome.

In this work, to investigate the structure of the EGFR/RAS/RIT1 signaling network and identify therapeutic targets in lung cancer, we perform genome-wide CRISPR screens in isogenic PC9 cells where drug resistance is potently conferred by each expressed oncogene. We find that the dependencies identified broadly confirm the expected pathway hierarchy but also identify key differences that highlight the importance of genotype-guided treatment stratification. A key difference we identify is in sensitivity to mitotic perturbation; *RIT1*- and *KRAS*-mutant cells differ in their sensitivity to Aurora kinase inhibitors due to a role of RIT1^M90I^ in the spindle assembly checkpoint. Furthermore, we identify YAP1 activation as a key cooperating event in *RIT1*-mutant lung cancer. In addition, we identify many other candidate *EGFR-*, *KRAS*-, and *RIT1* dependencies that should be further explored as drug targets for lung cancer therapy. This study expands our knowledge of EGFR/RAS signaling in lung cancer and provides a genome-wide discovery of factors that cooperate with or antagonize oncogenic RIT1.

## Results

### Context-specific differences in RIT1- and KRAS-stimulated ERK activity

**Fig. 1:**
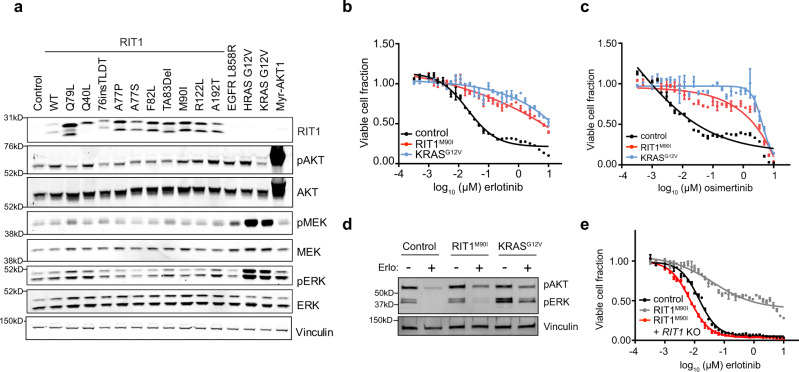
RIT1^M90I^ and KRAS^G12V^ promote resistance to EGFR tyrosine kinase inhibitors. **a** Western blot of lysates from NIH3T3 cells stably expressing a panel of RIT1 variants or mutant RAS, EGFR^L858R^, myristolated AKT1 (Myr-AKT1), or empty vector (control). Vinculin was used as a sample processing control. pAKT, phosphorylated Ser473 AKT1/2/3. pERK, phosphorylated Thr202/Tyr204 ERK1/2. pMEK, phosphorylated Ser217/221 MEK1/2. **b** Dose–response curve of 96-hour erlotinib treatment in isogenic PC9-Cas9 cells expressing the indicated oncogene or Firefly luciferase (control). CellTiterGlo was used to quantify viable cell number and viable cell fraction determined by normalization to DMSO control. Data shown are the mean ± s.e.m. of two technical replicates. **c** Dose–response curve of osimertinib, performed as in (**b**). Data shown are the mean ± s.e.m. of two technical replicates. **d** Western blot of lysates from cells shown in (**b**, **c**), cultured in the absence or presence of 500 nM erlotinib for 72 h. pAKT, phosphorylated Ser473 AKT1/2/3. pERK, phosphorylated Thr202/Tyr204 ERK1/2. Vinculin was used as a loading control. Blot representative of n = 2 independent experiments. **e** Dose–response curve of 96 h erlotinib treatment in clonal *RIT1* knockout (KO) cells compared to parental PC9-Cas9-RIT1^M90I^ cells or control PC9 cells. Data generated and presented as in (**b**). Data shown are the mean ± s.d. of two technical replicates. Unless otherwise indicated, all data are representative results from *n* = 2 independent experiments. Source data are provided as a Source Data file.

Our previous work found that cancer- and Noonan-associated RIT1 variants such as RIT1^M90I^ can transform NIH3T3 fibroblasts to a phenotype reminiscent of RAS-transformed cells^7^. However, when we examined cellular signaling by Western blot in the same cells, we discovered that oncogenic RIT1 variants fail to promote AKT, MEK, or ERK phosphorylation, whereas both KRAS^G12V^ and HRAS^G12V^ induce marked increases in MEK/ERK phosphorylation (Fig. 1a). Prior data showed that RIT1 variants are capable of robustly activating AKT, MEK, and ERK in PC6 pheochromocytoma cells^7^, but more subtly induce MEK/ERK phosphorylation in murine embryonic fibroblasts^15^, so we examined additional models to see if RIT1^M90I^ function varies in a cell-type dependent manner. Expression of RIT1^M90I^ in two different human lung epithelial cell lines, SALE or AALE^17^, did increase MEK phosphorylation, but the degree of stimulation differed between the two cell lines despite similar RIT1^M90I^ levels (Supplementary Fig. 1a). AKT phosphorylation was weakly induced in SALE cells only. These data suggest that context-dependent factors determine RIT1’s ability to stimulate MEK and AKT phosphorylation.

We previously demonstrated that RIT1^M90I^ and other RIT1 variants, as well as KRAS^G12V^, confer resistance to EGFR targeted therapy in *EGFR*-mutant lung cancer cells^8^. Expression of either RIT1^M90I^ or KRAS^G12V^ in PC9 *EGFR*-mutant lung adenocarcinoma cells renders them resistant to the EGFR inhibitors erlotinib (Fig. 1b) or osimertinib (Fig. 1c), conferring an almost 1000-fold decrease in drug sensitivity. Despite similar degrees of erlotinib- and osimertinib resistance in PC9-RIT1^M90I^ and PC9-KRAS^G12V^ cells, induction of ERK phosphorylation in *RIT1*-mutant cells was absent to low, while *KRAS*-mutant cells showed marked rescue of phosphorylated ERK levels (Fig. 1d). Phosphorylation of AKT was induced similarly in *RIT1*- and *KRAS*-mutant cells (Fig. 1d). We confirmed that resistance conferred by RIT1^M90I^ required continued expression of RIT1^M90I^ because erlotinib or osimertinib resistance could be reversed by knockout of *RIT1* using CRISPR-Cas9 technology (Fig. 1e, Supplementary Fig. 1b–d). These data indicate that stimulation of ERK phosphorylation by RIT1 is cell-type dependent and may involve distinct effectors than KRAS. This finding prompted us to develop a systematic approach to map the differing cellular consequences of oncogenic RIT1 and KRAS activation.

### A genome-scale platform for identification of oncogene-specific dependencies in lung cancer

Genome-scale CRISPR screens have been used to identify genetic dependencies of oncogenes in cancer, notably *KRAS*^18–21^. Comparative analyses of dependencies in mutant versus wild-type cell lines such as those from the Broad Institute Dependency Map^22^ are powerful resources for the discovery of oncogene vulnerabilities. However, the vast majority of mutated cancer genes occur in a relatively low fraction of tumors and cell lines. Indeed, only one lung adenocarcinoma cell line, NCI-H2110, has been identified with a canonical RIT1^M90I^ mutation^7^. An alternative strategy is CRISPR screens of isogenic cell models^20,23^ in which introduced oncogenes confer a selectable phenotype to the cells. Because RIT1 variants can confer resistance to erlotinib in PC9 cells, an *EGFR*-mutant lung cancer cell line^8^, this drug resistance phenotype could provide a powerful screening system to probe the requirements for RIT1 function in a highly controlled and robust system.

**Fig. 2:**
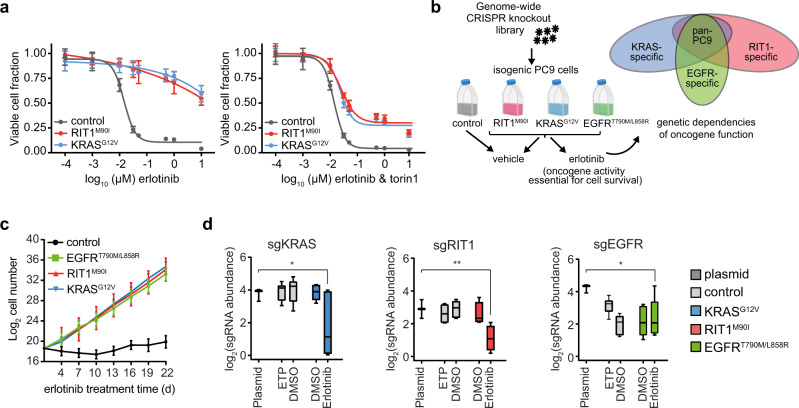
Building an integrative oncogene-dependency map of EGFR^T790M/L858R^, KRAS^G12V^, and RIT1^M90I^. **a** 96-hour dose–response of isogenic PC9 cells to erlotinib alone (left panel) or to a 1:1 molar ratio of erlotinib and torin1 (right panel). Fraction of viable cells was determined using CellTiterGlo and normalized to the average value in DMSO treated cells. Data shown are the mean ± s.d. of 8 technical replicates. **b** Schematic of the genome-wide CRISPR-Cas9 screens performed in isogenic PC9 cells. Created with BioRender.com. **c**, Proliferation rates of the PC9-Cas9 isogenic cells used for genome-wide screening in 50 nM erlotinib. Data shown are the mean ± 95% confidence interval for two replicates per cell line. **d** Box plot showing sgRNA abundance (log_2_ reads per million) of sgRNAs targeting each indicated gene. Abundance in the plasmid library, early time point (ETP), or after 12 population doublings in vehicle (DMSO) or erlotinib was determined by PCR and Illumina sequencing. Box plots show the median (center line), first and third quartiles (box edges), and the min and max range (whiskers) of replicates. * *p* = 0.022 (sgKRAS), ** *p* = 0.009 (sgRIT1), * *p* = 0.004 (sgEGFR) calculated by one-sided permutation testing using MAGeCK. For control PC9 cells ETP and DMSO, *n* = 3 biological replicates. For oncogene-expressing PC9 cells DMSO and erlotinib, *n* = 2 biological replicates. Source data are provided as a Source Data file.

As a proof of concept, we tested whether small-molecule inhibition of downstream signaling components of the EGFR/RAS pathway could overcome RIT1^M90I^-induced erlotinib resistance in PC9 cells. We ectopically expressed RIT1^M90I^ and KRAS^G12V^ in PC9 cells using lentiviral transduction and then cultured the cells in the presence of erlotinib or torin1, a small-molecule inhibitor of mTOR. Co-treatment with torin1 partially re-sensitized cells to erlotinib, demonstrating that both RIT1^M90I^ and KRAS^G12V^ likely act upstream of mTOR to induce erlotinib resistance (Fig. 2a).

To systematically define the factors required for RIT1^M90I^- and KRAS^G12V^-driven drug resistance we developed a pooled genome-wide knockout approach in drug-resistant PC9 cells (Fig. 2b). Isogenic pools of PC9 cells were generated expressing Cas9 and either a Firefly luciferase vector negative control (control), RIT1^M90I^, KRAS^G12V^, or EGFR^T790M/L858R^, an erlotinib-resistant mutant of EGFR used as a positive control (Supplementary Fig. 2a). Cas9 activity was confirmed by a sgGFP-GFP flow cytometry assay (Supplementary Fig. 2b, c). Long-term erlotinib resistance was assessed by culturing cells in the presence of 50 nM erlotinib for 22 days (Fig. 2c). Whereas cells expressing RIT1^M90I^, KRAS^G12V^, or EGFR^T790M/L858R^ all proliferated at a similar rate in erlotinib, control PC9 cells failed to expand in erlotinib (Fig. 2c).

To identify genetic dependencies required for PC9-Cas9-RIT1^M90I^, PC9-Cas9-KRAS^G12V^, and PC9-Cas9-EGFR^T790M/L858R^ cell proliferation in erlotinib, we used the Brunello CRISPR library consisting of 1000 non-targeting guides and 76,441 guides targeting 19,114 human genes^24^. Cells were transduced, selected with puromycin, and split to erlotinib or vehicle (DMSO) conditions, then maintained in erlotinib or DMSO for approximately 12 population doublings (Supplementary Fig. 2d). The change in abundance of each sgRNA was determined by sequencing and comparing the endpoint sgRNA abundance to the initial sgRNA abundance in the plasmid library (Supplementary Data 1), which was highly correlated with early time point (ETP) replicates taken just after lentiviral transduction and puromycin selection. Replicate screens were well-correlated (*R*^2^ range 0.58–0.94, Supplementary Fig. 2e) and exhibited expected lethality of known essential genes with a median strictly standardized mean difference (SSMD) of −3.9 (Supplementary Fig. 2f, Supplementary Data 1). To enable quantitative comparisons across screens, we adapted previously established methods^22^ to compute normalized CRISPR scores (CS), scaling the data such that the median CS of all sgRNAs targeting known essential genes is −1 and the median CS of those targeting known nonessential genes is 0 (Methods, Supplementary Data 2). As expected, expressed genes were, on average, more essential than non-expressed genes (Supplementary Fig. 2g). Importantly, as expected, sgRNAs targeting *KRAS* or *RIT1* were negatively selected in PC9-Cas9-KRAS^G12V^ and PC9-Cas9-RIT1^M90I^ cells, respectively, in erlotinib (Fig. 2d), validating the requirement of each oncogene for cell survival. As predicted, control PC9 cells are already dependent on *EGFR* for survival, so PC9-Cas9 cells expressing the erlotinib-resistant EGFR^T790M/L858R^ variant also remain dependent on *EGFR* in both erlotinib-treated and vehicle-treated conditions (Fig. 2d).

The concept of synthetic lethality originally referred to the phenomenon in which two genetic mutations cause a lethal phenotype whereas either mutation alone is tolerated. In cancer biology, the concept has been adapted to identify oncogene dependencies that might be leveraged for cancer therapy^25–27^. Such vulnerabilities could be directly related to the oncogene’s reliance on downstream effectors and pathways, or they might be created via indirect rewiring of cell phenotype. Here we define both baseline synthetic lethal knockouts and oncogene dependencies. We consider baseline synthetic lethal genes to be those whose knockout is lethal in the baseline DMSO treatment condition of oncogene-expressing cells but not vector control PC9 cells (Supplementary Data 3). In contrast, we define oncogene dependencies as those genes that are specifically required for the phenotype directly attributed to the oncogene–in this case, cell survival upon EGFR inhibition (Supplementary Data 4). It is reasonable that either baseline synthetic lethal or oncogene dependencies could be useful for therapeutic development in oncology, but here we focus specifically on oncogene dependencies because these are definitively linked to the assay phenotype. We expect many of these to also be essential for tumorigenesis induced by each oncogene (see below).

### No evidence of genetic interaction between KRAS and RIT1

Next, we sought to understand whether RIT1 and KRAS were required for drug resistance induced by each other. To this end, we evaluated whether knockout of *RIT1* underwent selection in KRAS^G12V^-mutant cells or vice versa. No significant change in abundance of *RIT1-* or *KRAS*-targeting sgRNAs was observed in the other cell line (Supplementary Fig. 2h, i), suggesting that *RIT1* and *KRAS* are not required for drug resistance conferred by the other gene. A similar result was seen for *RIT1* and *KRAS* sgRNAs in EGFR^T790M/L858R^-expressing cells, suggesting neither *RIT1* nor *KRAS* is required for drug resistance driven by EGFR^T790M/L858R^. Given the well-known role of RAS activation downstream of EGFR^28^, this result is surprising. However, it is thought that activation of PI3K in *EGFR*-mutant cells is sufficient for cell survival in erlotinib^29^. It is also possible that expression of KRAS paralogs HRAS and NRAS maintain RAS activation in the absence of KRAS, since paralog redundancy is increasingly recognized to influence CRISPR knockout phenotypes^30,31^.

Acquired *KRAS* mutations can drive EGFR inhibitor resistance in patients but are rarely observed^32^. The low rate of acquired *KRAS* mutations may be due to the mutual exclusivity of *KRAS* and *EGFR* mutations in primary lung adenocarcinoma^6,33^. Work in mouse models has shown that co-expression of mutant *KRAS* and *EGFR* is detrimental to cancer cells, leading to negative selection of cells co-expressing both oncogenes^34^. If true, knockout of *EGFR* should be positively selected in *KRAS*-mutant cells. In support of this model, KRAS^G12V^-mutant cells better tolerated loss of *EGFR* in erlotinib, maintaining high abundance of *EGFR* sgRNAs, in contrast to control PC9 cells or *EGFR-* or *RIT1*-mutant cells (Supplementary Fig. 2j).

### Integrative analysis of isogenic CRISPR screen data reveals oncogene-dependent biology

**Fig. 3:**
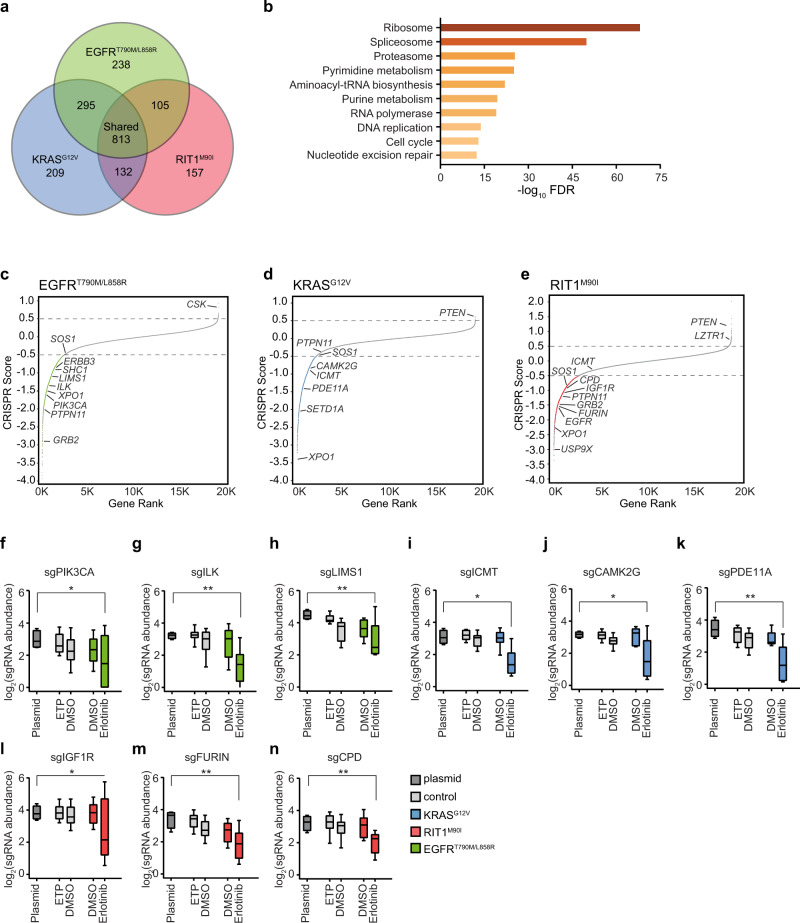
Genome-wide identification of EGFR^T790M/L858R^, KRAS^G12V^, and RIT1^M90I^ oncogene-specific genetic dependencies. **a** Venn Diagram showing the number of significant essential genes (CRISPR Score < 0.5 and *p* < 0.05) shared or specific to each isogenic PC9 cell line in erlotinib. **b** MSigDB overlap analysis of the enriched KEGG gene sets in the shared 813 genes from (**a**). **c**–**e**, Rank plots of CRISPR scores (CS) of erlotinib-treated vs. starting plasmid in (**c**), PC9-Cas9-EGFR^T790M/L858R^ (**d**), PC9-Cas9-KRAS^G12V^, and (**e**), PC9-Cas9-RIT1^M90I^. Key dependencies discussed in the text are labeled. Gray dashed lines mark genes with |CS| > 0.5. **f**–**n** Box plot showing the sgRNA abundance (log_2_ reads per million) of sgRNAs targeting each indicated gene. Abundance in the plasmid library, early time point (ETP), or after 12 population doublings in vehicle (DMSO) or erlotinib was determined by PCR and Illumina sequencing. Box plots show the median (center line), first and third quartiles (box edges), and the min and max range (whiskers) of replicates. **f**–**h** sgRNAs targeting *PIK3CA* (* *p* = 0.022), *ILK* (** *p* = 0.006*)*, or *LIMS1* (** *p* = 0.005) in control or PC9-Cas9-EGFR^T790M/L858R^ cells. **i**–**k** sgRNAs targeting *ICMT* (* *p* = 0.025), *CAMK2G* (* *p* = 0.045), or *PDE11A* (** *p* = 0.004) in control or PC9-Cas9-KRAS^G12V^. **l**–**n** sgRNAs targeting *IGF1R* (* *p* = 0.023), *FURIN* (** *p* = 0.003), or *CPD* (** *p* = 0.009) in control or PC9-Cas9-RIT1^M90I^ cells. * *p* < 0.05, ** *p* < 0.01, calculated by one-sided permutation testing using MAGeCK. For control PC9 cells ETP and DMSO, *n* = 3 biological replicates. For oncogene-expressing PC9 cells DMSO and erlotinib, *n* = 2 biological replicates. Source data are provided as a Source Data file.

To identify significantly enriched and depleted gene knockouts, we applied MAGeCK^35^, which identified an average of 64 positively selected gene knockouts and 1369 essential genes per cell line in erlotinib (|CS| > 0.5 and *p* < 0.05; Supplementary Data 2). Of these, 813 were shared across all three isogenic cell lines in erlotinib whereas 238, 209, and 157 were unique to *EGFR*, *KRAS*, and *RIT1*-mutant cells, respectively (Fig. 3a, Supplementary Data 4). As expected from prior studies^36^, pan-essential genes were significantly enriched for spliceosomal, ribosomal, and proteasomal genes (Fig. 3b).

Integration of data from the isogenic cell line screens enabled us to distinguish genes that are broadly essential in PC9 cells in erlotinib from gene knockouts that are negatively and positively selected in cells whose survival is driven by EGFR^T790M/L858R^ (Fig. 3c), KRAS^G12V^ (Fig. 3d), or RIT1^M90I^ (Fig. 3e), respectively. The landscape of these oncogene-specific dependencies differed in specific genes that recapitulated expected biology and pathway hierarchy. For example, in *EGFR*-mutant cells, top essential genes included known co-receptors and downstream pathway components *ERBB3*, *SHC1*, *GRB2*, *PIK3CA*, and *PTPN11*, also known as *SHP2* (Fig. 3c, f, Supplementary Fig. 3a–d). In addition to these well-characterized EGFR downstream components, we found that erlotinib resistance conferred by EGFR^T790M/L858R^ was uniquely dependent on integrin-linked kinase (*ILK*) and its binding partner *LIMS1/PINCH1* (Fig. 3g, h). In cancer, ILK and adaptor proteins regulate interactions between tumor cells and the extracellular environment to activate signaling pathways that promote cell proliferation, migration, and epithelial-to-mesenchymal transition^37^. In several human tumors, including non-small cell lung cancer, high ILK and LIMS1 expression correlates with increased disease progression^38,39^ and in *EGFR*-mutant patients, correlates with significantly worse progression-free survival after treatment with EGFR inhibitors^40^. Consistent with a role for ILK in mediating EGFR tyrosine kinase inhibitor (TKI) resistance, xenograft models of EGFR inhibitor-resistant human hepatocellular carcinoma cell lines found that inhibiting ILK activity increased the sensitivity of cells to EGFR inhibition^41^. Therefore, targeting ILK activity might be a valuable strategy for overcoming EGFR-TKI resistance in patients with EGFR^T790M/L858R^ mutations.

As predicted, *KRAS*-mutant cells were not reliant on upstream RTK signaling genes such as *ERBB3*, *SHC1*, or *GRB2* for survival in erlotinib (Supplementary Fig. 3a–c). Although the tyrosine phosphatase gene *PTPN11* has been recently identified as a potential therapeutic target in *KRAS*-mutant cancers^42^, *PTPN11* knockout did not significantly impact KRAS^G12V^-driven cell survival (Fig. 3d, Supplementary Fig. 3d). While this difference may be attributed to the different assay systems used, our finding is consistent with earlier reports showing that PTPN11 inhibition is lethal to cells with RTK activation but not to cells with oncogenic RAS proteins^43^. *RIT1-*mutant cells retained sensitivity to *PTPN11* depletion (Fig. 3e, Supplementary Fig. 3d), highlighting a functional divergence between RIT1^M90I^ and KRAS^G12V^. Instead, KRAS^G12V^-dependencies observed included *ICMT* (Fig. 3i), a methyltransferase responsible for the last step in a series of post-translational CAAX-domain modifications required for RAS to associate with the membrane^44^ and recently reported as a *KRAS* dependency^20^. RIT1 lacks a CAAX-domain and as predicted, *RIT1*-mutant cells did not rely on ICMT for survival in erlotinib (Fig. 3e). We observed enhanced sensitivity of KRAS^G12V^ cells to the previously reported *KRAS* dependency, *XPO1*^45^. However, loss of *XPO1* was lethal to all cell lines screened (Supplementary Fig. 3e). Additionally, we identified putative *KRAS* dependencies including *CAMK2G*, *PDE11A*, and *SETD1A* (Fig. 3j, k, Supplementary Fig. 3f).

Unlike *KRAS*-mutant cells, RIT1^M90I^-mutant cells were sensitive to knockout of RTK and adaptor protein genes including *PTPN11*, *GRB2*, and *EGFR* itself (Fig.  3e, Supplementary Fig. 3c, d). Moreover, RIT1^M90I^-mutant cells required insulin-like growth factor receptor 1 (*IGF1R*) (Fig. 3l) and several related components of IGF1R signaling. IGF1R is a multifunctional receptor that promotes cell proliferation, differentiation, and survival through activation of the PI3K-AKT and RAS/MAPK signaling pathways^46^. IGF1R is synthesized as an inactive precursor proprotein, which requires endoproteolytic cleavage to gain full biological activity^47,48^. In addition to IGF1R, *RIT1*-mutant cells were dependent on the proprotein convertase FURIN (Fig. 3m), and carboxypeptidase D (Fig. 3n), two proteins directly involved in IGF1R maturation and activity^49,50^.

To validate the genetic dependencies identified and determine the robustness of these screening results, we designed a custom sgRNA library consisting of 1000 non-targeting sgRNAs and 10,333 unique sgRNA sequences targeting 1288 genes (Methods, Supplementary Data 5). We performed secondary screening in three replicates each of vehicle- and erlotinib-treated PC9-Cas9-RIT1^M90I^ cells. Results from the primary screen and validation screen were highly correlated (*R*^2^ = 0.77; Supplementary Fig. 3g), with 75.4% of essential genes validating and 100% of positively selected genes validating in the secondary screen.

Together, these data illustrate the utility of CRISPR screens in isogenic cell lines for the identification of oncogene dependencies in cancer. Comparative analysis of EGFR^T790M/L858R^, KRAS^G12V^, and RIT1^M90I^ dependencies confirmed expectations from established pathway hierarchy. Moreover, the high validation rate in the secondary screen demonstrates the reproducibility of the majority of identified dependencies.

### Enhanced sensitivity of *RIT1*-mutant cells to loss of mitotic regulators

**Fig. 4:**
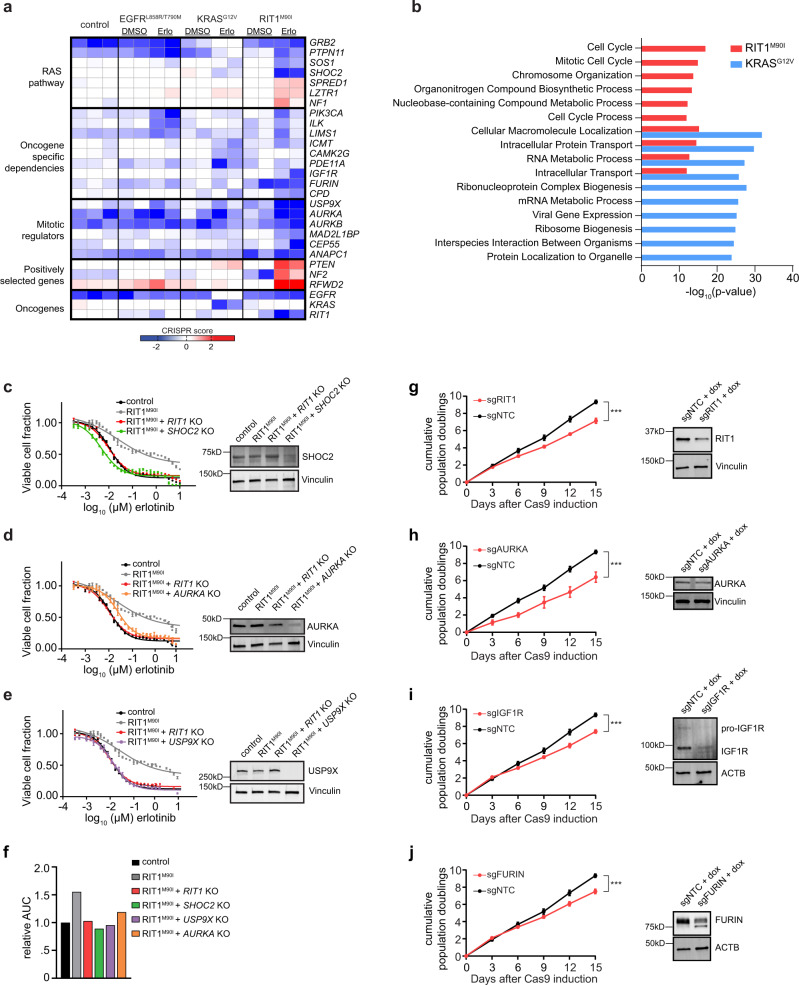
*RIT1*-mutant cells are vulnerable to loss of RAS pathway and cell cycle genes. **a** Heatmap illustrating the CS of selected dependencies clustered by biological pathway. Each column is a different replicate and shows CS between erlotinib or DMSO versus plasmid. **b** MSigDB overlap analysis of the GO Biological Process gene sets significantly enriched in the top 500 PC9-Cas9-RIT1^M90I^ and PC9-Cas9-KRAS^G12V^ essential genes. **c**–**e** Left, 96-hour dose–response curve of erlotinib in clonal *SHOC2* knockout (KO), *AURKA* KO, or *USP9X* KO cells derived from PC9-Cas9-RIT1^M90I^ cells. The same data for control and PC9-Cas9-RIT1^M90I^ and *RIT1* KO cells are plotted on each panel for reference. Data shown are the mean ± s.d. of two technical replicates. Right, Western blot for SHOC2, AURKA, or USP9X expression in clonal knockout cells derived from PC9-Cas9-RIT1^M90I^ cells. Vinculin was used as a loading control. **f** Area-under-the-curve (AUC) analysis of data from panels (**c**–**e**). **g-j** Left, Cumulative population doublings of H2110iCas9 cells grown in the presence of doxycycline (+dox) expressing the indicated sgRNAs compared to cells expressing non-targeting control sgRNA (sgNTC). The same data for sgNTC is shown on each panel for reference. Data shown are the mean ± s.d. of three technical replicates. *** *p* < 0.001 (**g** *** *p* = 0.0005**. h** *** *p* = 0.001. **i** *** *p* = 0.0003**. j** *** *p* = 0.0007.) by unpaired two-tailed t-test. Right, Western blot of day 5 lysates generated from parental H2110iCas9 or pooled *RIT1* KO, *AURKA* KO, *IGF1R* KO, or *FURIN* KO cells. Vinculin or Actin (ACTB) was used as a loading control. Source data are provided as a Source Data file.

Next, we sought to further investigate the different dependencies conferred by mutant *RIT1* and *KRAS*. *RIT1*-mutant cells were dependent on several known positive regulators of RAS signaling, including *SOS1* and *SHOC2* (Fig. 4a), while loss of negative regulators of RAS and genes commonly mutated in Noonan syndrome, such as *NF1*, *SPRED1*, and *LZTR1*, promoted RIT1-induced cell proliferation in erlotinib (Fig. 4a). In addition to regulators of RAS signaling, several *RIT1* dependencies were involved in mitotic spindle assembly and cell cycle regulation (Fig. 4a, b). A top *RIT1*-mutant dependency was *USP9X*, which encodes a deubiquitinase with many target substrates^51–53^ (Fig. 3e, Fig. 4a). During mitosis, USP9X plays an important role in regulating anaphase initiation and chromosome segregation by stabilizing key mitotic regulators such as Survivin and CDC20^51–54^. Knockout of *USP9X* was significantly depleted in RIT1^M90I^ cells in erlotinib, but not in KRAS^G12V^-mutant cells in erlotinib or control PC9 cells in DMSO (Fig. 4a). In addition to *USP9X*, *RIT1*-mutant cells were sensitive to depletion of mitotic regulators including *AURKA*, *AURKB*, and *MAD2L1BP*/*p31*^*comet*^ (Fig. 4a). Pathway enrichment analysis confirmed that cell cycle and mitotic regulators were significantly depleted in *RIT1*-mutant cells (Fig. 4b). This effect could conceivably be due to differences in proliferation rates; however, doubling rates were identical between the two cell lines (Fig. 2c). Secondary screening confirmed the enhanced sensitivity of *RIT1*-mutant cells to depletion of *USP9X* and *AURKA* (Supplementary Fig. 3g, Supplementary Fig. 4a, b) along with the RAS pathway and Noonan syndrome genes *SHOC2* and *SOS1* (Supplementary Fig. 3g, Supplementary Fig. 4a, b). To further validate the *RIT1*-specific dependency on *USP9X*, *SHOC2* and *AURKA*, we individually knocked out each gene in PC9-Cas9-RIT1^M90I^ cells (Supplementary Fig. 4c, Methods, Supplementary Table 1). Consistent with the pooled CRISPR screen results, individual loss of *SHOC2*, *AURKA*, or *USP9X* each resulted in restored sensitivity to erlotinib (Fig. 4c–f) and osimertinib (Supplementary Fig. 4d, e).

To determine whether these oncogenic *RIT1* dependencies extended to other cell contexts and in the absence of erlotinib treatment, we evaluated their function in NCI-H2110, a RIT1^M90I^-mutant non-small cell lung cancer cell line^7^. We introduced a doxycycline-regulated Cas9 (iCas9) construct^55^ into NCI-H2110 cells, and then introduced individual *RIT1*, *AURKA*, *IGF1R*, and *FURIN* sgRNAs into pools of H2110iCas9 cells (Methods, Supplementary Table 2). Sanger sequencing and ICE analysis^56^ after five days of Cas9 induction found that 28–52% of each cell pool contained an indel mutation (Supplementary Fig. 4f), which in all cases resulted in decreased protein expression (Fig. 4g-j). Supporting a requirement for *RIT1*, *AURKA*, *IGF1R*, and *FURIN* in the proliferation of *RIT1*-mutant lung cancer, knockout of each gene significantly reduced cell proliferation of H2110iCas9 compared to cells expressing a non-targeting control guide (sgNTC) (Fig. 4g-j). These data suggest that *RIT1* candidate dependencies identified through pooled genome-wide CRISPR screening may represent true dependencies in naturally occurring *RIT1*-mutant lung adenocarcinomas and point to a specific vulnerability of RIT1^M90I^-mutant cells to loss of mitotic regulators, particularly those involved in the spindle assembly checkpoint.

### *RIT1*-mutant cells are sensitive to Aurora kinase inhibition

**Fig. 5:**
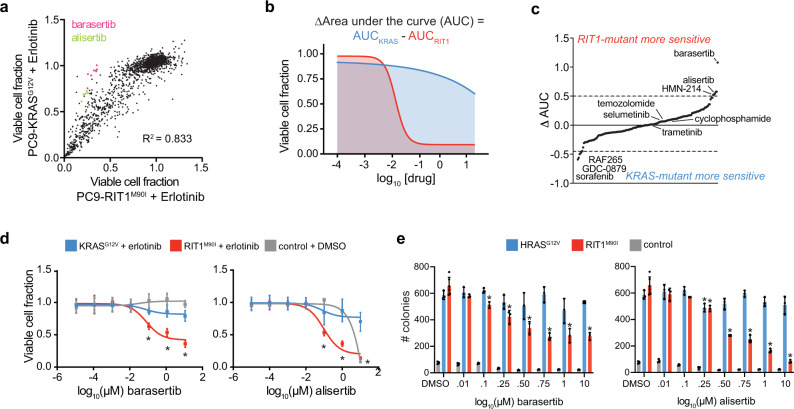
*RIT1*-mutant cells are sensitive to Aurora kinase inhibition. **a** Drug sensitivity screen of 160 small molecules, 8 doses per compound, in isogenic PC9-RIT1^M90I^ and PC9-KRAS^G12V^ treated in combination with each test condition and 500 nM erlotinib for 96-hours. Data shown are the mean of two replicates of each individual dose/compound. Viable cell fraction was calculated by normalizing CellTiterGlo luminescence in the test condition to CellTiterGlo luminescence of cells treated only with 500 nM erlotinib. **b** Schematic illustrating differential ‘area-under-the-curve’ (AUC) analysis. **c** ΔAUC analysis of 160 small molecules generated from data shown in (**a**) and calculated as in (**b**). Each dot represents a different compound. ΔAUC are median-centered and plotted from least to greatest. **d** Validation of enhanced response to barasertib, left, or alisertib, right. Data shown are mean ± s.d. of n = 16 replicates per dose. * *p* < 0.05 by unpaired two-tailed t-test. **e** Soft agar colony formation in NIH3T3 cells expressing RIT1^M90I^, HRAS^G12V^ or empty vector (control). Data shown are the mean ± s.d. of *n* = 3+ replicates for all conditions except for the 0.5 μM barasertib-treated RIT1^M90I^ cells (*n* = 2). * *p* < 0.05 by unpaired two-tailed t-test. Source data are provided as a Source Data file.

To further explore unique therapeutic vulnerabilities of *RIT1*-mutant cells, we used the same PC9 isogenic system to evaluate therapeutic efficacy of 160 small molecules, the majority of which are in clinical use or development. PC9-RIT1^M90I^ and PC9-KRAS^G12V^ were co-treated with erlotinib and each of the compounds at varying doses (eight per compound). The majority of compounds affected PC9-RIT1^M90I^ and PC9-KRAS^G12V^ cells with similar efficacy (*R*^2^ = 0.83; Fig. 5a, Supplementary Data 6). Comparison of the area under the curve (AUC) for each compound’s dose–response in each cell line (Fig. 5b) revealed that *RIT1*- and *KRAS*-mutant cells shared sensitivity to MEK inhibitors selumetinib and trametinib while sharing resistance to alkylating agents such as temozolomide and cyclophosphamide (Fig. 5c, Supplementary Data 6). A few compounds showed modest selectivity for *KRAS*-mutant cells, including three of the seven RAF inhibitors in the screen: sorafenib, RAF265, and GDC-0879, whereas top differentially sensitive compounds in PC9-RIT1^M90I^ cells were the Aurora kinase inhibitors alisertib, an inhibitor of Aurora A, and barasertib, an inhibitor of Aurora B (Fig. 5c). A PLK1 inhibitor, HMN-214 was also more effective in PC9-RIT1^M90I^ cells. Interestingly, Aurora A, Aurora B, and PLK1 are all important mitotic regulators with multifaceted functions including regulation of the spindle assembly checkpoint^57^.

We validated these findings not only in repeated PC9 drug response assays (Fig. 5d), but also in an independent cellular context, *RIT1*-mediated cell transformation in NIH3T3 cells. We found that both alisertib and barasertib treatment suppressed RIT1^M90I^-driven anchorage-independent growth, whereas transformation by oncogenic RAS was largely unaffected by Aurora inhibition (Fig. 5e). Together, these drug response and CRISPR genetic assays point to a unique sensitivity of *RIT1*-mutant cells to perturbation of mitotic regulators. Although genes such as Aurora kinases are generally essential in most cells^58^, our data suggest that *RIT1*-mutant cells may be unusually vulnerable to inhibition of these factors, which prompted us to investigate the mechanistic basis of this sensitivity.

### RIT1^M90I^ weakens the spindle assembly checkpoint

**Fig. 6:**
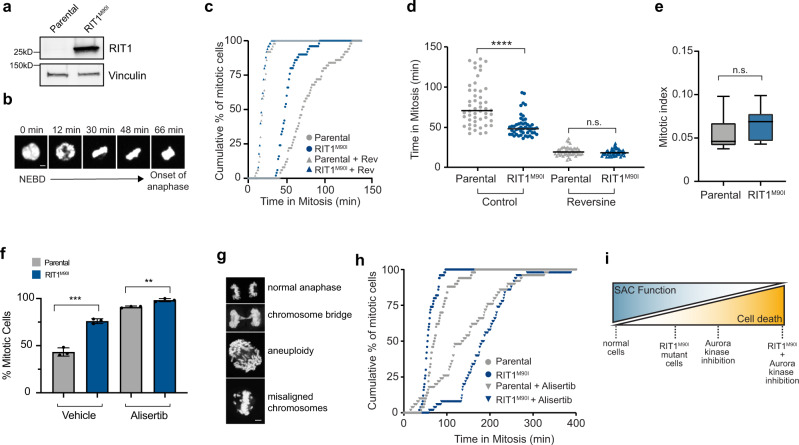
RIT1^M90I^ weakens the spindle assembly checkpoint. **a** Western blot of RIT1 expression in parental and RIT1^M90I^-expressing HeLa H2B-GFP cells. Vinculin was used as a loading control. **b** Duration of mitosis was measured as time from nuclear envelope breakdown (NEBD) to the onset of anaphase. Each frame represents movie stills from time-lapse live-cell imaging of parental HeLa H2B-GFP cells undergoing mitosis, scale bar = 5 µm. **c** Time-lapse fluorescence microscopy of time in mitosis in asynchronous parental and RIT1^M90I^-expressing HeLa H2B-GFP cells in normal media conditions (control) or treated with 0.5 µM reversine (Rev) two hours before imaging. *n* = 50 cells per condition. Mitotic timing was measured from time of NEBD to anaphase onset. **d** Alternative representation of data from (**c**). Data shown are the mean and individual data points of each condition. ****p = 9.06e^−9^, n.s., *p* = 0.1754 by unpaired two-tailed t-test. **e** Mitotic index calculated as the percentage of mitotic cells in a frame at a chosen time point. Box plots show the median (center line), first and third quartiles (box edges), and the min and max range (whiskers). *n* = 2 biological replicates, 6 time points per condition, n.s., *p* = 0.3587 by unpaired two-tailed t-test. **f** Comparison of mitotic error rates in HeLa H2B-GFP cells expressing RIT1^M90I^ compared to parental HeLa H2B-GFP cells in vehicle (DMSO)-treated and alisertib-treated (1 μM) conditions. *n* = 3 biological replicates, error bars indicate s.d., ***p = 0.0004, **p = 0.0029 by unpaired two-tailed t-test. **g** Representative images of mitotic errors from HeLa cells expressing RIT1^M90I^ and quantified in (**f**), scale bar = 5 µm. **h** Time-lapse fluorescence microscopy of time in mitosis in asynchronous parental and RIT1^M90I^-expressing HeLa H2B-GFP cells in normal media conditions   or treated with 1 µM alisertib two hours before imaging. *n* = 50 cells per condition. **i** Proposed model explaining enhanced efficacy of Aurora kinase inhibitors in RIT1^M90I^-mutant cells. Source data are provided as a Source Data file.

Fidelity of mitosis relies on the spindle assembly checkpoint (SAC), a critical cellular pathway that senses unaligned kinetochores and arrests mitotic progression during metaphase by inhibiting the anaphase-promoting complex/cyclosome (APC/C) until kinetochores are properly attached to microtubules^57^. Altering the SAC in normal cells accelerates mitotic timing^59^, and in conditions of mitotic stress can either promote mitotic cell death or result in mitotic slippage, the exit from mitosis before proper chromosome alignment is complete^60^. Given that many of the *RIT1* dependencies identified were components of the SAC (e.g. USP9X, Aurora kinases, MAD2L1BP), we hypothesized that RIT1^M90I^ might weaken the SAC, enhancing the vulnerability of the cells to further loss of SAC activity. To test this hypothesis, we adapted a model system commonly used for mitotic timing experiments^61^, HeLa cells expressing a nuclear H2B-GFP fusion protein^62^ (Fig. 6a). We used live-cell fluorescence microscopy to time the duration of mitosis from nuclear envelope breakdown (NEBD) to anaphase onset^59^ (Fig. 6b). In parental H2B-GFP cells the median duration of mitosis was 70.5 min (95% CI = 63–82 min), while in RIT1^M90I^-mutant cells the median duration of mitosis was reduced to 48 min (95% CI = 45–51 min) (Fig. 6c, d). Overall mitotic index was unaffected, suggesting that mitotic entry is not regulated by RIT1^M90I^ (Fig. 6e). The difference in mitotic timing between RIT1^M90I^-mutant cells and parental cells was eliminated by treatment with reversine, an inhibitor of the MPS1 kinase involved in establishing the SAC kinetochore signal, demonstrating that RIT1^M90I^ perturbs mitotic timing at the level of the SAC (Fig. 6c, d). A weakened SAC via perturbation of key components has been associated with mitotic errors including misaligned chromosomes, chromosome bridges, micronuclei formation, and aneuploidy^63–66^. Consistent with RIT1^M90I^ suppression of the SAC, *RIT1*-mutant cells showed significantly higher prevalence of chromosomal abnormalities compared to parental cells (Fig. 6f, g).

The vulnerability of *RIT1*-mutant cells to Aurora A inhibition and RIT1’s ability to weaken the SAC led us to hypothesize that Aurora A inhibition may be able to overcome the mitotic phenotype induced by RIT1^M90I^. To test this hypothesis, we performed mitotic timing analysis in HeLa H2B-GFP cells treated with the Aurora A inhibitor alisertib. Whereas RIT1^M90I^ alone accelerated mitosis compared to parental cells, mitotic timing in RIT1^M90I^ cells was increased compared to parental in the setting of alisertib (Fig. 6h). RIT1^M90I^-mutant cells accumulated more mitotic errors than parental cells in alisertib (Fig. 6f). Taken together, these data indicate that oncogenic RIT1 weakens the SAC, creating a vulnerability to Aurora kinase inhibitors. Because Aurora kinases are required for full activation of the SAC^53,67,68^, we propose a working model by which combined RIT1^M90I^ and Aurora A inhibition leads to cellular toxicity and cell death (Fig. 6i).

### YAP1 activation synergizes with RIT1^M90I^ to promote lung cancer

**Fig. 7:**
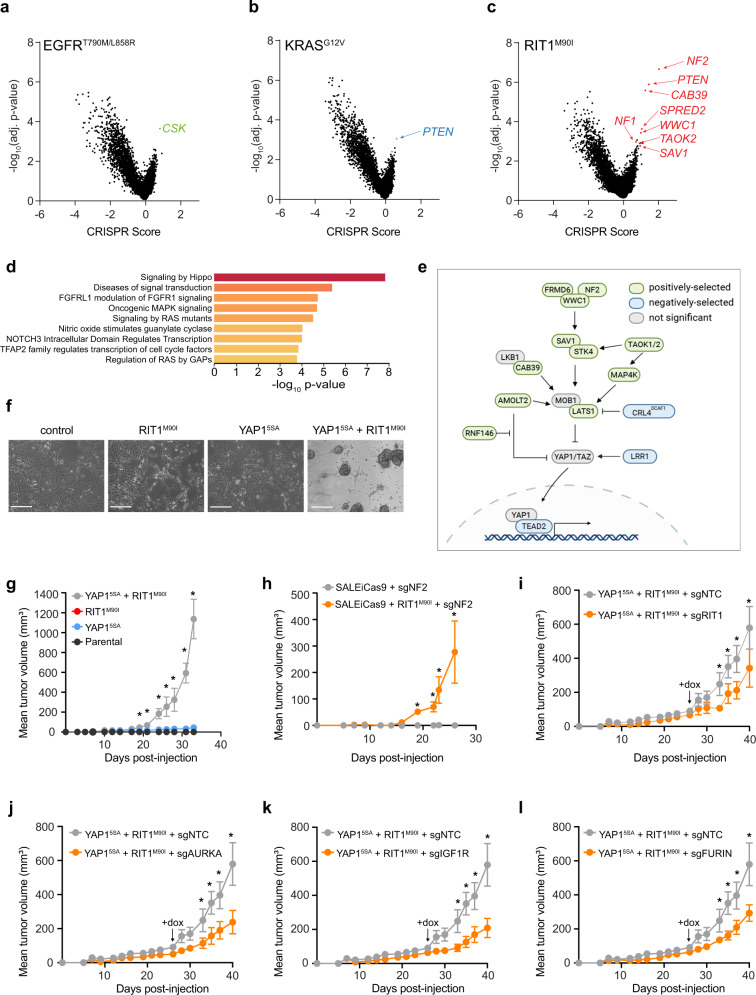
Hippo pathway inactivation synergizes with RIT1^M90I^ to promote cell survival and proliferation. **a**–**c** Volcano plots of genome-wide CRISPR screening data from (**a**) PC9-Cas9-EGFR^T790M/L858R^ cells, (**b**) PC9-Cas9-KRAS^G12V^ cells and (**c**) PC9-Cas9-RIT1^M90I^ cells cultured in 40 nM erlotinib for 12 population doublings. CRISPR Score indicates the normalized log_2_ (fold-change) of the average of 4 sgRNAs per gene in two biological replicates in erlotinib compared to the starting abundance in the plasmid library. **d** MSigDB overlap analysis of the top REACTOME gene sets in RIT1^M90I^ positively selected gene knockouts (*p* < 0.05, CRISPR score > 0.5). **e** Depiction of positively selected (green) and negatively selected (blue) Hippo pathway components identified in PC9-Cas9-RIT1^M90I^ (|CS| > 0.5 and *p* < 0.05). Schematic created partially with Biorender.com. **f** Phase contrast images of SALEiCas9 cells (control) or expressing RIT1^M90I^, the constitutively nuclear-localized YAP1^5SA^, or RIT1^M90I^ and YAP1^5SA^. Scale bar = 200 µm. Representative images from *n* = 3 independent experiments are shown. **g** Xenograft assay of SALEiCas9 cells expressing RIT1^M90I^, YAP1^5SA^, or combined RIT1^M90I^ and YAP1^5SA^ in immunocompromised mice. Data shown are the mean ± s.e.m. of *n* = 6 tumors per group. * *p* < 0.05 by unpaired two-tailed t-test. **h** Xenograft assay of SALEiCas9 cells expressing sgNF2, or combined RIT1^M90I^ and sgNF2 in immunocompromised mice. Data shown are the mean ± s.e.m. of n = 8 tumors per group. * *p* < 0.05 by unpaired two-tailed t-test. **i**-**l** Xenograft assays of SALEiCas9-RIT1^M90I^ + YAP1^5SA^ cells transduced with non-targeting control sgRNAs (sgNTC) or *RIT1*-, *AURKA*-, *IGF1R*, or *FURIN*-targeting sgRNAs (*n* = 8 tumors per sgRNA). Cells were pre-treated with doxcycyline in vitro for 5 days and re-induced at day 26 (arrow) in vivo with doxycycline-containing chow. * *p* < 0.05 by unpaired two-tailed t-test. Source data are provided as a Source Data file.

In addition to differences in genetic dependencies, each oncogene displayed substantial differences in the landscape of positively selected gene knockouts (Fig. 7a–c). *PTEN* knockout, which is known to promote erlotinib resistance^69,70^, cooperated with both KRAS^G12V^ and RIT1^M90I^ but showed no evidence of selection in EGFR^T790M/L858R^ cells (Supplementary Fig. 5a), consistent with EGFR’s ability to activate the PI3K pathway on its own. In contrast, drug resistance driven by EGFR^T790M/L858R^ was promoted by knockout of *CSK* (Fig. 7a), which encodes C-terminal SRC kinase, a negative regulator of SRC^71^. SRC activates EGFR via phosphorylation, to enhance EGFR kinase activity and prolong signaling by suppressing EGFR degradation^72^. Enrichment for *CSK* knockout was not observed in *KRAS-* or *RIT1*-mutant PC9 cells (Fig. 7b, c). Whereas *EGFR*-mutant and *KRAS*-mutant cells showed few cooperating events (n = 37 and n = 5 knockouts, respectively), RIT1^M90I^-mutant cells had 152 significantly enriched gene knockouts in erlotinib (Fig. 7c). Five of the top 12 genes whose loss synergized with RIT1^M90I^ belong to the Hippo pathway: *NF2*, *CAB39*, *WWC1*, *TAOK2*, and *SAV1* (Supplementary Fig. 5b-f). Among all positively selected gene knockouts (*p* < 0.05, CS > 0.5), pathway enrichment analysis using the Molecular Signatures Database (MSigDB) identified the Hippo signaling pathway as the top REACTOME pathway (FDR = 2.08e-5) (Fig. 7d, e). Secondary screen validation confirmed robust enrichment of Hippo pathway knockouts including *NF2*, *CAB39*, and *TAOK1* in RIT1^M90I^-mutant cells (Supplementary Fig. 3g, Supplementary Fig. 5g). Validation experiments using pooled and clonal knockout cells demonstrated that loss of *NF2* cooperated with RIT1^M90I^ to promote erlotinib resistance (Supplementary Fig. 5h, i).

The Hippo pathway is a tumor-suppressive cellular signaling pathway that regulates proliferation and cell survival via negative regulation of YAP1 stability and nuclear translocation^73^. Enrichment for loss of Hippo pathway genes in RIT1^M90I^ cells suggests that inactivation of Hippo signaling may synergize with oncogenic RIT1 to promote cellular proliferation and survival. However, YAP1 activation has previously been shown to promote erlotinib resistance itself^74^, so we tested whether YAP1 activation synergizes with RIT1^M90I^ in the absence of erlotinib using a human small airway lung epithelial (SALE) cell transformation model^8^. To test whether RIT1^M90I^ or activated YAP1 can transform these cells, we expressed RIT1^M90I^ alone or in combination with YAP1^5SA^, which harbors five serine-to-alanine mutations at critical LATS1/2 phosphorylation sites, resulting in a stabilized, nuclear-localized YAP1 protein^75^. The co-expression of RIT1^M90I^ and YAP1^5SA^ in SALE cells caused the cells to shift from an adherent to a suspension growth phenotype (Fig. 7f, Supplementary Fig. 5j). Moreover, co-expression of RIT1^M90I^ and YAP1^5SA^ synergistically transformed SALE cells to induce xenograft tumor formation (Fig. 7g, Supplementary Fig. 5j). Similar results were obtained by knocking out *NF2* in combination with RIT1 expression in SALE cells (Fig. 7h, Supplementary Fig. 5k). Together these data demonstrate that oncogenic RIT1 and activated YAP1 cooperate to promote tumorigenesis.

Next, we leveraged the finding that RIT1^M90I^ and YAP1^5SA^ synergize to promote tumorigenesis in SALE cells to expand the validation of oncogenic RIT1 dependencies *AURKA*, *IGF1R,* and *FURIN* in vivo. We introduced doxycycline-inducible Cas9 into SALE cells co-expressing RIT1^M90I^ and YAP1^5SA^ (RIT1^M90I^ + YAP1^5SA^) followed by transduction with sgNTC, sgRIT1, sgAURKA, sgIGF1R, or sgFURIN constructs. Doxycycline-treatment of xenografts of RIT1^M90I^ + YAP1^5SA^ xenografts resulted in significant inhibition of tumor growth compared to sgNTC control (Fig. 7i-l, Supplementary Fig. 5l-o). These data extend the cellular contexts in which these RIT1 dependencies are found to be critical for RIT1 function and confirm that these genes are relevant not only for RIT1-driven drug resistance but also for tumorigenesis.

### YAP1 activation and downregulation of Hippo pathway genes in human *RIT1*-mutant lung cancer

**Fig. 8:**
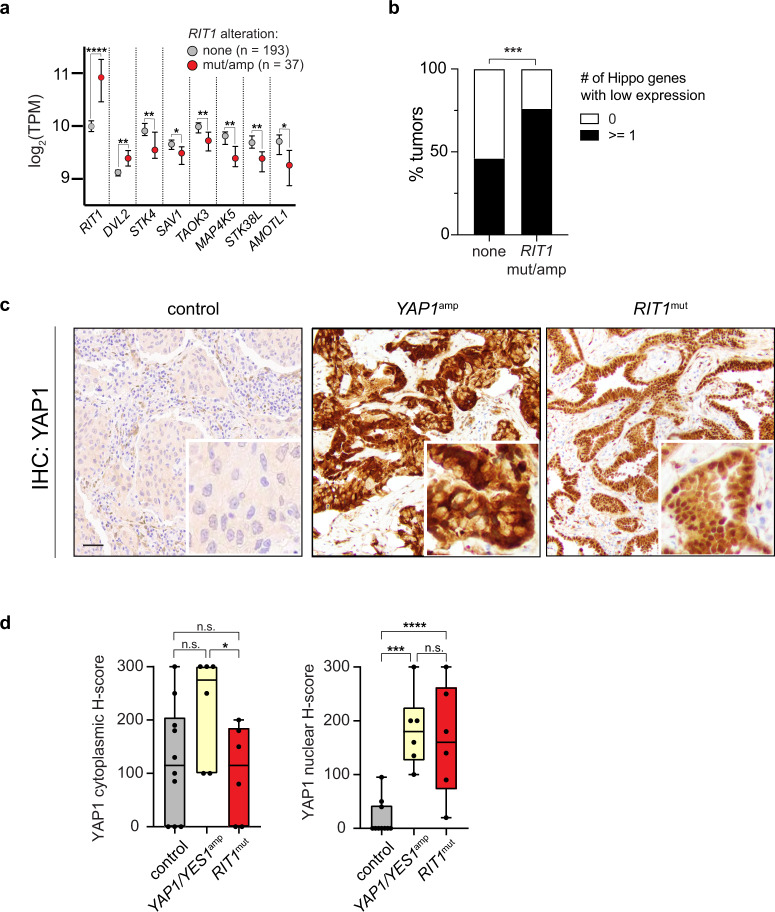
Hippo pathway loss and YAP1 nuclear overexpression in RIT1-mutant human lung tumors. **a** RNA-sequencing data of human lung adenocarcinomas from TCGA. Data shown are the median ± 95% confidence interval of log_2_-transformed transcripts per million (TPM) in *RIT1*-altered (amplified or mutated, *n* = 37) tumors compared to *RIT1* non-altered tumors (*n* = 193). * *p* < 0.05, ** *p* < 0.01, **** *p* < 0.0001 by unpaired two-tailed t-test (*RIT1:* **** *p* = 1.84e−11, *DVL2*: ** *p* = 0.0025, *STK4*: ** *p* = 0.0011, *SAV1*: * *p* = 0.0278, *TAOK3*: ** *p* = 0.0052, *MAP4K5*: ** *p* = 0.0023, *STK38L*: ** *p* = 0.0023, *AMOTL1*: * *p* = 0.0384). **b** Proportion of control or* RIT1* amplified or mutated (mut/amp) tumors with low expression of any one Hippo pathway gene; see Methods. *** *p* = 0.0006 by one-tailed Fisher’s exact test. **c** Representative low magnification images and inset zoom of YAP1 IHC in human lung tumor samples, control, *YAP1*-amplified, and *RIT1*-mutant, scale bar = 100 µm. *n* = 6–10 tumors per condition were analyzed **d** H-score quantification of control, *YAP1/YES1*-amplified, and *RIT1*-mutant human lung tumor samples (Supplementary Data 8). H-Score = % of positive cells multiplied by intensity score of 0–3; see Methods. *n* = 6–10 tumors per condition were analyzed. Box plots show the median (center line), first and third quartiles (box edges), and the min and max range (whiskers). n.s., *p* > 0.05, * *p* = 0.046, *** *p* = 0.0009, **** *p* < 0.0001 by unpaired two-tailed t-test. Source data are provided as a Source Data file.

To investigate whether co-activation of YAP1 and RIT1 occurs in human lung cancer, we analyzed data from 230 human lung adenocarcinomas sequenced by The Cancer Genome Atlas^6^. Because wild-type *RIT1* overexpression can transform cells^8^ and may confer Noonan syndrome in individuals with germline *LZTR1* mutations^15^, we included *RIT1*-amplified tumors in our analysis. 16% of lung adenocarcinomas harbored mutated or amplified *RIT1* (Supplementary Fig. 6a). Tumors showed copy number-related increases in *RIT1* mRNA expression, indicating that *RIT1*-amplified tumors overexpress *RIT1* (Supplementary Fig. 6b). Analysis of differentially expressed genes in *RIT1*-mutant and *RIT1*-amplified lung adenocarcinomas identified significant loss of expression of Hippo pathway genes (FDR = 6e−4). Driving this enrichment was the downregulation of key Hippo pathway genes *STK4*, *SAV1*, *TAOK3*, *MAP4K5*, *STK38L*, and *AMOTL1* (Fig. 8a). Other significantly altered pathways included EGFR inhibitor resistance (FDR = 2.09E−6), PI3K-AKT (FDR = 2.09E−6), and MAPK signaling (FDR = 7.6E−4) (Supplementary Data 7). In contrast, the WNT-pathway gene *DVL2* was overexpressed (Fig. 8a), which may further activate YAP1 via crosstalk between WNT and Hippo signaling networks^76,77^. 76% (28/37) of *RIT1*-altered tumors had low expression of at least one Hippo pathway gene, compared to 46% (88/193) of tumors with wild-type/normal *RIT1* (Fig. 8b; ***p < 0.001 by one-sided Fisher’s exact test).

To confirm this observation in an independent clinical cohort, we identified *RIT1*-mutant non-small cell lung tumors using MSK-IMPACT sequencing^78^ (Supplementary Data 8). We performed YAP1 immunohistochemistry (IHC) comparing YAP1 localization in *RIT1*-mutant tumors to tumors with known YAP1 activation by chromosomal amplification of *YAP1* or *YES1*^79^, or control tumors with wild-type *RIT1* and *YAP1/YES1* (Fig. 8c). When Hippo signaling is suppressed, YAP1 becomes unphosphorylated and translocates to the nucleus to regulate transcription^80^. Indicative of YAP1 activation, *RIT1*-mutant tumors had significantly higher YAP1 nuclear H-scores than control tumors, similar to that seen in *YAP1*-amplified tumors (Fig. 8c, d). Together, these data show that Hippo inactivation synergizes with mutant *RIT1* in cancer models, and Hippo pathway inactivation and YAP1 activation also occurs in *RIT1*-altered human lung tumors.

To investigate the molecular basis for the observed functional synergy, we performed RNA-seq in SALE cells expressing RIT1^M90I^ and YAP1^5SA^ individually or in combination (Supplementary Fig. 6c–e, Supplementary Data 9). Interestingly, RIT1^M90I^ and YAP1^5SA^ induced very different transcriptional states with little correlation (Supplementary Fig. 6c, left panel). Transcripts altered by RIT1^M90I^ were not enriched for a YAP1 gene signature^81^, whereas YAP1^5SA^ drove activation of this signature as expected (Supplementary Fig. 6d). However, when RIT1^M90I^ and YAP1^5SA^ were co-expressed, YAP1-regulated gene expression was enhanced by RIT1^M90I^ compared to YAP1^5SA^ alone (Supplementary Fig. 6c, e), with known YAP1 targets such as *TNNT2*, *ITGB2*, and *COL4A3* showing 2.5-3.6 fold increased expression compared to YAP1^5SA^ alone (Supplementary Fig. 6e), which was confirmed by qRT-PCR (Supplementary Fig. 6f) Although the specific mechanism of RIT1 and YAP1 synergy remains to be fully elucidated, these data suggest that RIT1^M90I^ on its own does not trigger YAP1 target transcription but may instead enhance activation of YAP1 targets in settings of YAP1 activation, such as tumors with Hippo pathway loss.

## Discussion

Activation of the RTK-RAS signaling pathway is a near-universal hallmark of lung adenocarcinoma^1^, and drug-targetable mutations identified within the pathway have been harnessed to develop genotype-guided therapies. While targeted therapies sometimes result in exceptional tumor responses and have improved lung cancer patient survival, selection for tumor cell adaptation occurs rapidly, leading to acquired resistance and necessitating treatment with second- and third-line targeted agents until eventual treatment failure^82^. Highlighting the key requirement for activation of RTK-RAS signaling, acquired resistance usually occurs via second-site mutations in the original oncogene or bypass activation of alternative signaling molecules in the network. Although each mutated oncogene in this network shares the ability to activate this pro-tumorigenic cellular signaling pathway, the overlapping but distinct signaling mechanisms of each protein create unique cellular vulnerabilities.

The role of *RIT1* activation is a less well-understood part of the RTK-RAS signaling pathway in lung cancer. In this work, we used genetic dependency mapping to uncover the shared and distinct functional requirements of oncogenic variants of *EGFR*, *KRAS*, and *RIT1*. An advantage of our experimental model system was the specific reliance of the cells on the activity of the expressed oncogene in erlotinib, a phenotype firmly linked to each introduced mutation. By intersecting the common dependencies across isogenic cell lines, we were able to exclude pan-essential genes and genes that generally alter sensitivity/resistance to EGFR inhibition, an approach similar to the Daisy Model of gene essentiality^83^. This strategy allowed us to overcome the challenging lack of patient-derived experimental model systems in which to study mutant RIT1 function. A disadvantage of the experimental system, however, was its reliance on treatment with erlotinib, which would not typically be used for the treatment of *RIT1*-mutant lung cancers. It was therefore critical to validate the dependencies in other cell models in the absence of erlotinib. To this end, we were fortunate to identify a model of *RIT1*-mutant tumorigenesis through our identification of RIT1/YAP1 synergy in the transformation of human lung epithelial cells (Fig. 7g-l).

RIT1’s involvement in Noonan syndrome and lung adenocarcinoma has confirmed its role in the RAS signaling pathway, although the specific mechanism of its function in this pathway has remained elusive. Although RIT1 is reported to physically interact with C-Raf^15^, its ability to activate ERK appears limited compared to RAS and is cell-type dependent (Fig. 1a, d and ref. ^15^.). In contrast, oncogenic RAS has been studied for decades and is thought to act downstream of the SHP2/PTPN11 phosphatase and SOS1 guanine nucleotide exchange factor to activate effectors including RAF, PI3K, and RalGDS^84^. In agreement with this model, our data show that RAS-mutant cells are insensitive to knockout of *SOS1* and *PTPN11* (Fig. 4a). However, other studies suggest that inhibition of PTPN11 can suppress RAS-driven tumorigenesis^42,85^, so further research is required to understand the cell types and contexts where PTPN11 inhibition may be effective. Surprisingly, we find that RIT1^M90I^ differs from KRAS^G12V^ in its dependency on these factors as well as on the SHOC2 scaffolding protein involved in RAF activation (Fig. 4a). *RIT1*-mutant cells depend on *PTPN11*, *SOS1*, and *SHOC2*, while showing no requirement for *KRAS* itself. Likewise, in *KRAS*-mutant cells, *RIT1* is not required, suggesting KRAS and RIT1 play distinct roles in the regulation of this pathway. An additional difference between *RIT1*- and *KRAS*-mutant cells is the continued reliance of *RIT1*-mutant cells on RTK complex proteins such as GRB2 and IGF1R. Combined with the requirement for *PTPN11* and *SOS1*, we speculate that RIT1^M90I^ may be involved in the activation of RTKs themselves, or drive feedback signaling to RTKs. Further studies are needed to clearly define the structure of RIT1’s requirement for these proteins.

Beyond these classic RTK-RAS signaling components, we uncovered a surprising vulnerability of *RIT1*-mutant cells to perturbation of mitotic regulators, particularly components of the spindle assembly checkpoint. We found that *RIT1*-mutant cells showed heightened sensitivity to loss of mitotic regulators such as *AURKA, USP9X, MAD2L1BP*, and *PLK1*, whether by genetic inactivation or small-molecule inhibition, despite no differences in cell proliferation or mitotic index. We showed that RIT1^M90I^ weakens the spindle assembly checkpoint, leaving the cells vulnerable to Aurora kinase inhibitors. While our manuscript was in review, a preprint was posted showing the discovery of RIT1 as a MAD2-binding protein that inhibits the mitotic checkpoint complex to accelerate mitotic timing^86^. Using a similar mitotic timing assay to that presented here, they showed that oncogenic RIT1^M90I^ accelerates mitosis in U2-OS and HeLa cells. One difference was that the assay was performed in nocodazole; otherwise, the result is nearly identical to the data we show in HeLa cells, suggesting that regulation of mitotic timing and induction of mitotic errors are a general function of oncogenic RIT1. The study, now published^87^, also showed that wild-type RIT1 participates in the spindle assembly checkpoint and that knockout of endogenous *RIT1* extends mitotic timing in multiple cell types. Therefore, RIT1 normally participates in the spindle assembly checkpoint and pathogenic levels of RIT1^M90I^ alter this normal regulation. The results of our study further imply that this mitotic phenotype confers a targetable vulnerability to *RIT1*-mutant cells, particularly in the context of Aurora kinase inhibition. Continued studies should investigate additional cell line and cancer models to understand the basis and prevalence of RIT1^M90I^-driven alisertib sensitivity. Future genotype-directed clinical trials could determine if patients with *RIT1*-mutant tumors would uniquely benefit from treatment with Aurora kinase inhibitors or other modulators of mitosis. Interestingly, Aurora A activation has been found to drive resistance to the third-generation EGFR inhibitor osimertinib^88^. It is possible that RIT1^M90I^ is harnessing this same mechanism to drive erlotinib resistance and cellular transformation.

One of the strongest genetic interactions identified was the pronounced synergy between loss of Hippo pathway genes and *RIT1* mutation. The ability of activated YAP1 or *NF2* loss to cooperatively transform human lung epithelial cells with RIT1^M90I^ (Fig. 7g, h) suggests that this synergy occurs not only in the context of drug resistance, but more generally in oncogenesis. Our analysis of human tumor data showed that over 75% of *RIT1*-altered lung adenocarcinomas harbor co-occurring loss of expression of at least one Hippo pathway gene, and we verified high rates of YAP1 nuclear staining in an independent *RIT1*-mutant tumor cohort. While the precise mechanism underlying this synergy remains to be determined, multiple lines of evidence suggest that RIT1^M90I^ and YAP1 may be acting independently to cooperatively transform cells. First, mutant *RIT1* did not directly stimulate a YAP1 transcriptional signature in RNA-seq of RIT1^M90I^-expressing cells (Supplementary Fig. 6d). Second, we observed strong positive selection for loss of upstream Hippo pathway genes; if RIT1 was already promoting Hippo inactivation or YAP1 activation, this cooperation would not be expected. We observed this cooperative effect across multiple different models and phenotypes: loss of *NF2* synergized with RIT1^M90I^ in the PC9 erlotinib resistance assay and promoted tumor formation of human SALE cells in vivo. The latter phenotype was also conferred by YAP1^5SA^, suggesting that the effect of Hippo suppression is to promote nuclear localization of YAP1. The finding of nuclear YAP1 staining in human *RIT1*-mutant tumors suggests that RIT1 variants may be most oncogenic in the context of YAP1 activation, and therefore targeting YAP/TEAD activity should be explored as a strategy to suppress *RIT1*-mutant lung cancer. Future efforts to dissect the mechanism of RIT1/YAP synergy and determine the sensitivity of *RIT1*-mutant cells to YAP inhibitors should provide further insight into the cooperation between these two oncogenes.

More broadly, our work demonstrates the utility of genome-wide CRISPR screens in isogenic cell lines to identify oncogene dependencies and discover therapeutic targets. Isogenic cell systems are particularly valuable to identify critical dependencies of oncogenes that are mutated in <5% of cases and consequently not well-represented in CRISPR screening databases such as DepMap^22,89^. The use of deeply characterized phenotypes that are closely linked to and predictive of oncogene function is essential. These directed approaches will complement the analysis of larger cell line panels towards the goal of building genome-scale dependency maps for all human oncogenes.

## Methods

### Cell Lines

PC9 cells were a gift from Dr. Matthew Meyerson (Broad Institute) and NCI-H2110 cells were obtained from ATCC (CRL-5924). PC9 and NCI-H2110 were cultured in RPMI-1640 (Gibco) supplemented with 10% Fetal Bovine Serum (FBS). NIH3T3 cells were obtained from ATCC (CRL-1658), and HeLa-H2B cells were a gift from Dr. Daphne Avgousti (Fred Hutchinson Cancer Research Center) and were originally acquired from Millipore (SCC117). HEK293T cells were obtained from ATCC (CRL-3216). HEK293T, NIH3T3, and HeLa-H2B cells were cultured in Dulbecco’s Modified Eagle’s Medium (DMEM, Genesee Scientific) supplemented with 10% FBS (Sigma). AALE and SALE human lung epithelial cells are immortalized with hTERT and the early region of SV40 and were a gift of Dr. William Hahn (Dana-Farber Cancer Institute) and were cultured in small airway epithelial growth media (SAGM) with SAGM supplements and growth factors (PromoCell or Lonza). All cells were maintained at 37 °C in 5% CO2 and confirmed mycoplasma-free.

### Lentivirus production

Lentivirus was produced using standard triple transfection protocols: HEK293T cells were co-transfected with lenti-vector (pLKO/PLX303/PLX317/PXPR003), pCMV-VSV-G (Addgene no. 8454) and psPAX2 (Addgene no. 12260) in TransIT-LTI (Mirus Bio) and OptiMEM (Thermo Fisher Scientific). 18 h post-transfection, media was changed to high-serum DMEM (30% FBS). Lentivirus was harvested 48 h post-transfection.

### Vector construction and cell line generation

For NIH3T3 signaling analysis and soft agar assays, the following plasmids were obtained from Addgene; pDONR223-HRAS^G12V^ (Addgene no. 82090), pDONR223-EGFR^L858R^ (Addgene no. 82906), pDONR223-KRAS^G12V^ (Addgene no. 81665), and pBabe-puro-Myr-FLAG-AKT1 (Addgene no. 15294). pBabe-puro *RIT1* plasmids were generated in a former study^7^. Isogenic NIH3T3 were generated by retroviral transduction and selection with 2 µg/ml puromycin. Protein expression was confirmed by Western blotting. For cell signaling analysis in SALE and AALE cells, pDONR223-KRAS^WT^ (Addgene no. 81751), pDONR223-KRAS^G12V^, and pDONR223-RIT1^M90I^ cDNAs were recombined into pLX317 lentiviral expression vector and lentivirus was generated as above. SALE and AALE cells were transduced with lentivirus followed by selection with 1.5 µg/ml puromycin. Protein expression was confirmed by Western blotting.

For the whole-genome CRISPR knockout screen, PC9 cells stably expressing Cas9 were generated by transducing cells with Cas9 pXPR_111 lentivirus (Genetic Perturbation Platform, Broad Institute) and selecting with blasticidin for 5–7 d. Cas9 protein expression was determined by Western blot. To determine Cas9 activity, PC9-Cas9 cells were transduced with a pXPR_011-sgEGFP (Addgene no. 59702), a vector encoding both GFP and a sgRNA targeting GFP. Following selection with puromycin cells were expanded for 3 days. In parallel, untransfected parental PC9 cells and parental PC9 cells transfected with only pXPR_011-sgEGFP were maintained and used as controls for non-GFP expressing and GFP expressing cells, respectively. GFP expression was analyzed in all three cell lines by flow cytometry and data was analyzed using FlowJo software v10.6.2 (Tree Star Inc, Stanford).

To generate isogenic PC9-Cas9 cell lines, pDONR233 vectors encoding EGFR^T790M/L858R^ (Addgene no. 82914), KRAS^G12V^ (Addgene no. 81665), RIT1^M90I^ (described above), and Firefly luciferase (Addgene no. 25894) were subcloned into the pLX317 lentiviral expression vector. Following verification by Sanger sequencing and restriction digest, stable PC9-Cas9 isogenic cell lines were generated by lentiviral transduction in the presence of 2 µg/ml polybrene followed by selection with 250 µg/mL hygromycin for 48-72 h. Stable lines were expanded, and protein expression was confirmed by Western blotting.

For the small-molecule screen, PC9-RIT1^M90I^ and PC9-KRAS^G12V^ were generated by transducing parental PC9 cells with lentivirus generated from pLX317-RIT1^M90I^ and pLX317-KRAS^G12V^ generated as described above.

To generate H2110iCas9 cells, NCI-H2110 cells were transduced with lentivirus generated from Lenti-iCas9-neo (Addgene no. 85400) in the presence of 2 µg/ml polybrene followed by selection with 400 µg/mL G418/Geneticin^TM^ (ThermoFisher Scientific, 10131035).

To generate isogenic SALEiCas9 cells, parental SALE and SALE-RIT1^M90I^ cells were transduced with Lenti-iCas9-neo (Addgene no. 85400) and selected with 400 µg/mL G418/Geneticin^TM^ (ThermoFisher Scientific, 10131035). *YAP1*^*5SA*^ cDNA was PCR amplified from pQCXIH-Myc-YAP1-5SA (Addgene no. 33093) and cloned via Gibson Assembly^90^ into the lentiviral FU-CGW vector^91^ which expresses GFP. SALEiCas9 and SALEiCas9 RIT1^M90I^ cells were transduced with YAP1^5SA^ lentivirus and 48 h post-transduction GFP expression was confirmed by direct fluorescent expression using the EVOS FL digital inverted microscope Cell Imaging System v1.4 (Thermo Fisher Scientific).

### Genome-wide CRISPR gene knockout screen

The human CRISPR Brunello lentiviral pool was obtained from the Broad Institute Genetic Perturbation Platform and is also available from Addgene (73179-LV). The library contains 76,441 sgRNAs targeting 19,114 protein-coding genes and 1,000 non-targeting control sgRNAs. For genome-wide CRISPR screening, 320 million PC9-Cas9-Luciferase, PC9-Cas9-RIT1^M90I^, PC9-Cas9-KRAS^G12V^, or PC9-Cas9-EGFR^T790M/L858R^ cells were infected with the Brunello Library lentivirus^24^ at a low MOI (<0.3). At 24 h after infection, the medium was replaced with fresh media containing 1 μg/mL puromycin (Sigma). After selection on day 7, cells were split into 2 or 3 replicates containing 40 million cells each and treated with either DMSO (Sigma-Aldrich) or 40 nM erlotinib (Selleckchem). Cells were then passaged every 3 days and maintained at 500-fold coverage. For early time point analysis (day 7) an initial pool of 60 million cells was harvested for genomic DNA extraction from each of the cell lines. After ~12 doublings, a final pool of 60 million cells was harvested in ice-cold PBS and stored at −80°.

Genomic DNA was extracted using the QIAamp DNA Blood Maxi Kit (QIAGEN) and the sgRNAs from each sample were PCR amplified by dividing gDNA into multiple 100 μl reactions containing a maximum of 10 μg gDNA (as recommended by Broad Institute standard protocols). Per 96-well plate, a master mix consisted of 150 μl ExTaq polymerase (Takara Bio), 1000 μl of 10x ExTaq buffer (Takara Bio), 800 μl of dNTP (Takara Bio), 50 μl of P5 primer (stock at 100 μM concentration), and 2,075 μl water. Each well consisted of 50 μl gDNA plus water, 40 μl PCR master mix, and 10 μl of P7 primer (stock at 5 μM concentration). Primer sequences are listed in Supplementary Table 3. PCR cycling conditions: an initial 5 min at 95 °C; followed by 30 s at 95 °C, 30 s at 53 °C, 20 s at 72 °C, for 28 cycles; and a final 10 min extension at 72 °C. PCR samples were purified with Agencourt AMPure XP SPRI beads (Beckman Coulter). Samples were sequenced on a HiSeq 2500 (Illumina). Raw FASTQ files were demultiplexed and sgRNA counts were calculated using PoolQ v2.2.0.

### CRISPR validation library and screening

For secondary screening, we generated a custom library containing 1000 non-targeting control sgRNAs and 11,333 sgRNAs targeting 1288 protein-coding genes (Supplementary Data 5). For validation screening, 180 million PC9-Cas9-RIT1^M90I^ were infected with the validation library lentivirus at a low MOI (< 0.3). At 24 h after infection, the media was replaced with fresh media containing 1 μg/mL puromycin (Sigma). After selection on day 7, cells were split into 3 replicates containing 6.6 million cells each and treated with either DMSO (Sigma-Aldrich) or 40 nM erlotinib (Selleckchem). Cells were then passaged every 3 days and maintained at 500-fold coverage. For early time point analysis (day 7) an initial pool of 20 million cells were harvested for genomic DNA extraction from each of the cell lines. After ~12 doublings, a final pool of 20 million cells were harvested for genomic DNA extraction using the QIAamp DNA Blood Maxi Kit (QIAGEN). PCR and sequencing were carried out as described above.

### Single and pooled gene knockout generation and genotyping

To induce *RIT1*, *NF2*, *USP9X*, *AURKA*, or *SHOC2* gene knockout in PC9-Cas9-RIT1^M90I^ cells, a vector-free CRISPR-mediated editing approach was used: cells were co-transfected using lipofectamine CRISPR max (Life technologies) with three gene-specific synthetic guide RNAs (Synthego, Supplementary Table 1).

To generate *RIT1, AURKA, FURIN*, and *IGF1R*, gene knockout in H2110iCas9 and SALEiCas9 cells, sgRNAs (Supplementary Table 2) were cloned into pLentiGuide-Puro (Addgene no. 52963). Lentivirus for each sgRNA was generated as described above. H2110iCas9 were transduced with sgRNA lentivirus in the presence of 2 µg/ml polybrene followed by selection with puromycin (2 μg/mL). SALEiCas9 cells were transduced with sgRNA lentivirus followed by selection with 1.5 µg/ml puromycin.

For each single-gene knockout cell line, gene editing was confirmed by Sanger sequencing. Custom oligos flanking the targeted sites were used to amplify genomic DNA from pooled edited cells (Supplementary Tables 4, 5) using High-Fidelity 2 × Master Mix (New England Biolabs). Indel frequencies were quantified by comparing unedited control and knockout cell lines using Inference of CRISPR Edits (ICE)^56^.

### Proliferation assay

H2110iCas9 cells expressing either *RIT1, AURKA, FURIN*, or *IGF1R* sgRNA or non-targeting control sgNTC were seeded in triplicate in 6-well dishes at a density of 0.25 × 10^6^ cells per well. After 24 h the media was replaced and supplemented with 1 µg/ml of doxycycline to induce Cas9 expression. At this point, cells were counted and passaged every 3 d and replated at a density of .25 × 10^6^ cells per well. For Indel analysis and protein expression cell pellets were collected on day 2 and day 5 for gDNA and protein extraction.

### Cell lysis and immunoblotting

Whole-cell extracts for immunoblotting were prepared by incubating cells on ice in RTK lysis buffer [20 mM Tris (pH 8.0), 2 mM EDTA (pH 8), 137 mM NaCl, 1% IGEPAL CA-630, 10% Glycerol] plus phosphatase inhibitors (Roche) and protease inhibitors (cOmplete, Mini, EDTA-free, Roche) for 20 min. Following centrifugation (>16,000 *g* for 15 min), protein lysates were quantified using the Pierce BCA Protein Assay Kit (Thermo Fisher Scientific). Lysates were separated by SDS–PAGE and transferred to nitrocellulose or PVDF membranes using the Trans-blot Turbo Transfer System (BioRad) or iBlot (Invitrogen). Membranes were blocked in 1x Tris Buffered Saline (TBS) with 1% Casein (BioRad) for 1 h at room temperature followed by overnight incubation at 4 °C with primary antibodies diluted in blocking buffer. StarBright (BioRad) or IRDye (LiCOR) secondary antibodies were used for detection and were imaged on ChemiDoc MP Imaging System (BioRad). Loading control and experimental protein(s) were probed on the same membrane unless indicated otherwise. For clarity, loading control is cropped and shown below experimental condition in all panels regardless of the relative molecular weights of the two proteins.

Primary antibodies used for immunoblotting: Phospho-p44/42 MAPK 1:500-1:1000 (Erk1/2) (Thr202/Tyr204) (Cell Signaling Technology, 4370), p44/42 MAPK 1:500-1:1000 (Erk1/2) (Cell Signaling Technology, 9107), Phospho-MEK1/2 1:500-1:1000 (Ser217/221) (Cell Signaling Technology, 9154), MEK1 1:500-1:1000 (Cell Signaling Technology, 2352), Phospho-AKT 1:500-1:1000 (Cell Signaling Technology, 4060), AKT 1:500 (Cell Signaling Technology, 2920), EGFR 1:500 (Cell Signaling Technology, 2239), β-Actin 1:1000 (Cell Signaling Technology, 4970), Vinculin 1:1500 (Sigma, V9264); Firefly Luciferase (Abcam, Ab16466), RIT1 1:1500 (Abcam, Ab53720), KRAS 1:500 (Sigma, WH0003845M1), NF2 1:1000 (Abcam, Ab109244), USP9X 1:1000 (Proteintech, 55054-1-AP), SHOC2/Sur-8 1:100 (Santa Cruz Biotechnology, sc-514779) IGF1R 1:1000 (Cell Signaling Technology, 9750), FURIN 1:500 (Thermo Scientific, PA1-062) and AURKA 1:500 (Cell Signaling Technology, 4718).

### Drug treatment and proliferation analysis

For proliferation assays cells were plated in 384-well plates at a density of 800 cells per well in 40 µl total volume. One day later, a serial dilution of each inhibitor was performed using a D300e dispenser (Tecan). 96 h post-treatment, cell viability was determined using CellTiterGlo reagent (Promega) and luminescence quantified on an Envision MultiLabel Plate Reader (PerkinElmer). To calculate the fraction cell viability drug-treated cells were normalized to average cell viability of DMSO-only treated cells. Curve fitting was performed using GraphPad Prism v9.1.1 four-parameter inhibitor response with variable slope. AUC values were calculated by GraphPad Prism v9.1.1. Inhibitors were obtained from SelleckChem: Erlotinib-OSI-744 (S1023), Osimertinib-AZD9291 (S7297), Torin 1 (S2827), Alisertib-MLN8237 (S1133), Barasertib-AZD1152 (S1147). DMSO (Sigma-Aldrich).

### Small-molecule drug screen

PC9-RIT1^M90I^ and PC9-KRAS^G12V^ were plated in 384-well plates at a density of 800 cells per well in 40 µl total volume. One day after seeding, cells were treated with 500 nM erlotinib in combination with the small-molecule library (a kind gift of Dr. Stuart Schreiber, Broad Institute). Each small molecule was tested across an 8-point dilution series (Supplementary Data 6). 96-hours post-treatment cell viability was determined using CellTiterGlo reagent (Promega) and luminescence quantified on an Envision MultiLabel Plate Reader (PerkinElmer). To calculate the fraction of cell viability, drug-treated cells were normalized to 500 nM erlotinib only treated cells. Dose–response curves were plotted using GraphPad Prism v9.1.1; AUC values were generated using GraphPad Prism v9.1.1 and three-parameter inhibitor response setting. Delta-AUC was calculated by subtracting the AUC for each compound in RIT1^M90I^ from the AUC of the same compound in KRAS^G12V^ cells. Each experiment was carried out in two technical replicates.

### Soft agar assays

For soft agar assay, 5 × 10^3^ NIH3T3 cells expressing RIT1^M90I^, HRAS^G12V^, or control (empty vector) were suspended in 1 ml of 0.33% select agar in DMEM/FBS and plated on a bottom layer of 0.5% select agar in DMEM/FBS in six-well dishes. Each cell line was analyzed in triplicate. Colonies were photographed after 14–21 days and quantified using CellProfiler v3.0^92^. For soft agar inhibitor experiments, alisertib (MLN8237) or barasertib (AZD1152) was suspended in the top agar solution at a final concentration of 0.01–10 μM.

### In vivo xenograft studies and ethical approval

All animal experiments were carried out with approval by and in accordance with the ethical guidelines of the Fred Hutchinson Cancer Research Center Institutional Animal Care and Use Committee (Protocol #50967, PI: Berger). Experiments were performed in the Fred Hutch Comparative Medicine facility, which is fully accredited by the Association for Assessment and Accreditation of Laboratory Animal Care (AAALAC) and complies with all United States Department of Agriculture (USDA), Public Health Service (PHS), Washington State and local area animal welfare regulations. Data and methods are reported here in accordance with ARRIVE guidelines^93^. All animals were housed in individually ventilated and HEPA-filtered microisolator cage environments (Allentown Inc. & Tecniplast) using reusable caging that is autoclaved prior to use and the Steam Plant Facility uses sterile, disposable caging. Additional housing information is as follows: light cycle 12:12 light: dark, temperature 72° ± 3 F, and humidity 30–70%. All animal feed and cage enrichment material is sterilized, and only purified, acidified water is provided. Husbandry is done in height adjustable HEPA-filtered laminar flow cabinets using high-level disinfectants. Athymic nude (nu/nu) mice (4 to 6 weeks old males) for the xenograft study were obtained from Jackson Laboratory, Bar Harbor, ME, USA and allowed to acclimate to the facility for at least 1 week. Due to a supplier shortage, females were not available for use. For SALE doxycycline-inducible Cas9 studies in RIT1^M90I^ + YAP1^5SA^ cells (Fig. 7i-l), cells were grown in culture and treated with 1 µg/ml doxycycline for five days prior to subcutaneous injection into the flanks of immunocompromised (NU/J) mice. SALE-iCas9 cells expressing sgNF2, RIT1^M90I^ + sgNF2, RIT1^M90I^ + YAP1^5SA^ + sgNTC, sgRIT1, sgAURKA, sgIGF1R, or sgFURIN were harvested by trypsinization, washed in PBS and resuspended at 10^6^ cells/ml in PBS. Two hundred microliters (2 × 10^6^ cells) were injected into each injection site, *n* = 2 injection sites/mouse; 4 mice/condition for 8 experimental replicates per cell line where each experimental unit is a single tumor/injection site. The sample size was chosen to give 98% power to detect a difference in means of 10% assuming standard deviation of 5%, or 98% power to detect a difference in means of 20% given a standard deviation of 10%. There were no exclusion or inclusion critera; all data points were used for analysis. Animals were not randomized; to avoid error animals within the same cage were injected with the same cell solution and likewise received the same cell solution at each site. Investigators were not blinded to the experimental group information. Cells were allowed to engraft for 1 week, then tumors were measured every 2–3 days using a digital caliper (VWR, Radnor, PA, USA). At day 26, mice were put on doxycycline chow ad libitum (625 ppm) and tumors were measured every 2–3 days until the largest tumor reached 2 cm in diameter. Tumor volume was calculated with the formula 0.5 × *L* × W^2^ where *L* is the longest diameter and *W* is the diameter perpendicular to *L*. Data were analyzed in GraphPad Prism (v9.1.1) and statistical significance was determined by multiple unpaired two-tailed t-tests at each time point for each experimental group compared to the sgNTC control with no multiple hypothesis correction and a p value less than 0.05 considered significant.

### Mitotic timing and chromosomal aberration analysis

RIT1^M90I^-expressing HeLa H2B-GFP cells were generated by transduction with a pLX303-RIT1^M90I^ lentivirus and selection with puromycin. Protein expression was confirmed by Western blotting. One day before imaging, cells were seeded at a density of 30,000 cells per well of an 8-well Ibidi glass-bottomed plate.  For drug-treated populations, 0.5 μM reversine (Selleckchem) or 1 µM alisertib was added two hours before imaging. Live-cell imaging was performed using a ×20/0.70 Plan Apo Leica objective on an automated Leica DMi8 microscope outfitted with an Andor CSU spinning disk unit equipped with Borealis illumination, an ASI automated stage with Piezo Z-axis top plate, and an Okolab controlled environment chamber (humidified at 37 °C with 5% CO_2_). Long-term automated imaging was driven by MetaMorph software (v7.10.0.119). Images were captured with an Andor iXon Ultra 888 EMCCD camera. Images were captured every minute for 18 h. Time in mitosis was measured as time from nuclear envelope breakdown to the onset of anaphase. Imaging experiment was repeated four times with distinct biological replicates and 50 cells were analyzed per cell line per condition.

Mitotic index was calculated from one time point selected from live-image experiments in HeLa H2B-GFP parental and HeLa H2B-GFP-RIT1^M90I^ cells described above. At each time point, three independent, representative images were collected and the mitotic index was calculated based on the number of cells undergoing mitosis (in any phase between prometaphase to telophase) divided by the total number of cells. The same time point was analyzed in two independent experiments for a total of 6 total mitotic index analyses per cell line.

For the mitotic abnormality analysis, HeLa H2B-GFP cells were plated in a 4-well Nunc^TM^ Lab-Tek^TM^chamber slide (ThermoFisher Scientific) at a density of 80,000 cells per well. The next day, cells were treated with either DMSO vehicle or 1 μM alisertib for 6 h prior to being washed with 1X PBS and fixed with 4% paraformaldehyde (from 16% paraformaldehyde [wt/vol]) in 1X PBS for 30 min at RT. Coverslips were mounted with Vectashield antifade mounting medium with 1.5ug/mL DAPI (Vector Laboratories). Slides were analyzed using a ×40/1.25 Oil PL Apo Leica objective on a Leica DMi8 outfitted with a TCS SPE scan head with spectral detection. Cells were visualized using the LAS X software platform (v3.5.7.23225). Representative images were captured using a ×40/1.30 Plan Apo Leica objective on a Leica DMi8 outfitted with a TCS SPE scan head with spectral detection. Images were acquired using the LAS X software platform (v3.5.5.19976). Images were corrected for brightness and contrast using FIJI (v2.1.0/1.53c). Images are single sections. For each biological replicate, 60 cells were analyzed per cell line. The prevalence of chromosome bridges, lagging and chromosome misalignment, micronuclei, aneuploidy (i.e. notable, unequal separation of chromosomes), polyploidy (i.e. evidence of unsuccessful cytokinesis) and normal separation of chromosomes were recorded.

### Gene set enrichment analysis

In Fig. 4b and Fig. 7d, gene set overlap analysis was performed using the MsigDB (v7.4)^94,95^ and StringDB (v11.0)^96^ databases. For all analyses an FDR cut-off of < .05 was used. To account for differences in the number of cooperating factors between RIT1^M90I^ and KRAS^G12V^, overlap analysis was carried out on the top 152 positively selected genes from each screen. In Supplementary Fig. 6d, GSEA of transcriptome data was completed using preranked gene lists using GSEA 4.1.0^90^ using the oncogenic signature gene sets (c6.all.v7.2.symbols.gmt) containing 189 gene sets. Number of permutations was set to 1000, classic enrichment statistic was used with 15 and 500 set as minimum and maximum, respectively, for set inclusion.

### TCGA RNA expression analysis

*RIT1* RNA-seq data from TCGA lung adenocarcinomas (230 samples)^6^ was obtained from cBioPortal (v3.6.18) for Cancer Genomics (http://cbioportal.org). The z-score for *RIT1* mRNA expression is determined for each sample by comparing *RIT1* mRNA expression to the distribution of *RIT1* expression in all diploid samples^97^.

For differential gene expression analysis, *RIT1*-altered samples were defined as tumors with either mutation of *RIT1* (*n* = 5) or amplification of *RIT1* (*n* = 32). The remaining 193 tumors were considered non-*RIT1*-altered. Differential expression analysis was performed in cBioPortal, and genes with *q*-value < = 0.25 were downloaded from cBioPortal (http://cbioportal.org). Pathway enrichment analysis was carried out on the 1015 under-expressed genes identified in *RIT1-*altered sample using the StringDB^96^ database with an FDR < .05.

For analysis of individual Hippo pathway genes in *RIT1* altered samples, we included the following genes as Hippo pathway genes: *AMOTL1*, *NF2*, *STK4*, *STK38L MAP4K5*, *TAOK3*, *SAV1*. To determine the portion of *RIT1* altered tumors with low expression of any one Hippo pathway gene, low expression was defined as >1 standard deviation lower than the mean expression of the 230-sample dataset. Then we determined the proportion of samples with low expression of at least one of the above genes, or with normal expression of all genes.

### YAP1 immunohistochemistry in *RIT1*-mutant human tumors

Epitope retrieval was performed using Leica Bond III ER2 for 30 min on the stainer, followed by incubation with the YAP1 antibody (Santa Cruz, clone 63.7) for an additional 30 min, and detection using Bond Polymer Refine DAB IHC detection kit. For quantitative assessment of YAP1 labeling in the nuclear and cytoplasmic compartments, a histoscore (H-score) was derived by multiplying percent positivity in tumor cells by the corresponding staining intensity (1 = weak, 2 = moderate, 3 = strong) within each compartment yielding a range of possible H-scores of 0 − 300.

### Transcriptome profiling

Three technical replicates per cell line were harvested at ∼90% confluence. Total RNA was extracted from lysed cells using Direct-zol RNA Miniprep plus (Zymo Research). Libraries of 50 bp paired-end reads were constructed using the Illumina TruSeq kit, which utilizes non-strand-specific poly-A selection. Libraries were pooled and sequenced on the Illumina HiSeq 2500 platform (Fred Hutch Genomics Core). Reads were aligned to the human reference genome build hg19/GRCh37 using STAR v2.5.3a^98^. Alignments were annotated, reordered, and indexed using Picard Tools v1.114 (http://broadinstitute.github.io/picard). To ensure sequencing and alignment quality, read statistics for each RNA-seq sample were calculated using RseQC v3.0.1^99^, including total library size, number of ribosomal RNA reads, and number of mapped and unmapped read pairs. Transcripts were quantified based on hg19 gene annotations using the featureCounts program included in Subread v1.5.3^100^. Gene-level CPM and RPKM values were calculated with edgeR v3.30.3^101^ then converted into transcripts per million values with an in-house script. In total, 12,458 genes were identified with average expression level of at least 0.1 log_2_CPM across all samples. Differential expression analyses comparing SALE-RIT1^M90I^, SALE-YAP1^5SA^ or SALE-RIT1^M90I^ + YAP1^5SA^ cells against vector control SALE were performed using edgeR v3.30.3^101^.

### RT-qPCR

Total RNA was extracted from three biological replicates of SALE parental, SALE-RIT1^M90I^, SALE-YAP1^5SA^ and SALE-RIT1^M90I^ + YAP1^5SA^ cells as described above. Reverse transcription (RT) was performed with 800 ng RNA and the SuperScript IV First-Strand Synthesis System (Invitrogen). For each quantitative RT-PCR reaction, input cDNA was optimized to the following amounts: 10 ng for *18S*, 40 ng for *ITGB2* and *COL4A3*, and 100 ng for *TNNT2* reactions. cDNA was amplified using TaqMan gene expression assays (ThermoFisher Scientific): *ITGB2* (assay ID: Hs00164957_m1), *TNNT2* (assay ID: Hs00943911_m1), *COL4A3* (assay ID: Hs01022502_m1), and *18S* (assay ID: Hs99999901_s1). Reactions were run using the BioRad CFX384 Real-Time system. Expression was normalized to 18S within each sample in the same experiment, and relative expression was quantified via the 2^-ΔΔCt^ method. Results are means of three technical replicates. Graphs and statistical analysis were performed in GraphPad Prism v9.1.1.

## Statistical analysis

### Genome-wide CRISPR screen

A log_2_-normalized count matrix of sequencing reads mapped to sgRNAs was used as an input to MAGeCK (v0.5.7)^35^. sgRNAs with < 1 read-per-million in the sequencing of the Brunello library plasmid were excluded from the dataset. The following sgRNAs were determined to not target the introduced cDNA oncogenes, and so were excluded from the dataset.

KRAS: (AGATATTCACCATTATAGGT) RIT1: (CATGCGGGACCAGTATATGA,GTGATGATCTGGCTTACCAA) EGFR: (TGTCACCACATAATTACCTG)

Reproducibility of the screen was assessed by calculating Pearson correlations of pairwise replicate comparisons for each replicate set (*n* = 2–3 replicates per screen arm). Pearson correlations ranged from 0.58 to 0.94 (median = 0.82). Scores from early time-point (ETP) samples were highly correlated to plasmid DNA (median = 0.93; Supplementary Fig. 2e) so comparison to plasmid DNA was used for all subsequent analyses. Guide RNAs were collapsed to gene scores using MAGeCK (v0.5.7), and log_2_ (fold-change) values (LFC) were computed compared to starting guide RNA abundance in plasmid DNA. To check the performance of each screen, we calculated robust strictly standardized mean difference (SSMD) statistics^22^ for each replicate, comparing the LFC between non-targeting sgRNAs and LFC values of sgRNAs targeting the spliceosomal, ribosomal, and proteasomal gene sets from KEGG^102^. All endpoint replicates passed the quality control threshold of SSMD < −0.5, with the median SSMD = −3.9 (Supplementary Data 1). Normalization across screens was based on published methods;^22^ first, the LFC data were normalized within each replicate by subtracting the median LFC of the replicate and then dividing by the median average deviation. Next, we scaled each replicate based on the LFC of common essential and nonessential genes (Supplementary Data 10) using previously defined lists by DepMap^89^. The data were scaled such that the median of the nonessential genes in each replicate is 0 and the median of the essential genes is −1. CRISPR scores were defined as this scaled, normalized LFC data. The final normalized, scaled data are supplied as (Supplementary Data 2). For analysis of expressed vs. non-expressed genes (Supplementary Fig. 2g), we defined expressed genes from RNA-sequencing data of PC9 cells^103^ as genes with log_2_(TPM) > 2 and non-expressed genes as log_2_TPM < 1.

### Statistical tests

Statistical tests are indicated in the figure legends. Results were analyzed for statistical significance with GraphPad Prism v9.1.1 or R (v3.6.3). A *p*-value of < 0.05 was considered significant (*p < 0.05, **p < 0.01, ***p < 0.001, ****p < 0.0001).

### Reporting summary

Further information on research design is available in the Nature Research Reporting Summary linked to this article.

## Supplementary information

Supplementary Information

Description of Additional Supplementary Files

Supplementary Data 1

Supplementary Data 2

Supplementary Data 3

Supplementary Data 4

Supplementary Data 5

Supplementary Data 6

Supplementary Data 7

Supplementary Data 8

Supplementary Data 9

Supplementary Data 10

Reporting Summary

## Data Availability

The RNA-seq data generated in this study have been deposited in the Gene Expression Omnibus under accession code GSE165631. Analysis of this dataset (Supplementary Fig. 6) is available in Supplementary Data 9. CRISPR screen data (Figs. 2–4, Supplementary Figs. 2–5) generated in this study are provided in Supplementary Data files as follows: sequencing results (Supplementary Data 1), normalized CRISPR scores (Supplementary Data 2), baseline synthetic lethal genes (Supplementary Data 3), oncogene dependencies (Supplementary Data 4), and validation screen analysis (Supplementary Data 5). The small-molecule screen data (Fig. 5) generated in this study are provided in Supplementary Data 6. Pathway analysis of TCGA RNA-seq (Fig. 8a) is provided in Supplementary Data 7. MSK-IMPACT information and H-scores (Fig. 8) is provided in Supplementary Data 8. Lists of common essential and non-essential genes from DepMap.org used for normalization of CRISPR data are provided in Supplementary Data 10. RNA expression data generated by TCGA were downloaded via cBioPortal for Cancer Genomics from http://www.cbioportal.org/study/summary?id=luad_tcga_pub. Source TCGA data used are available via Open Access at https://gdc.cancer.gov/about-data/publications/pancanatlas. The authors declare that all other data supporting the findings of this study are available within the paper and its Supplementary information files. Source data are provided with this paper, including uncropped gels for all Western blots and underlying data for all figures.

## Source data

Source Data
