# Supplementary material for: Integrative oncogene-dependency mapping identifies RIT1 vulnerabilities and synergies in lung cancer
Source: Nat Commun. 2021 Aug 9;12:4789. doi: 10.1038/s41467-021-24841-y (PMC8352964; doi:10.1038/s41467-021-24841-y)
Supplement: Supplementary file 1 — Supplementary Information [file 41467_2021_24841_MOESM1_ESM.pdf]

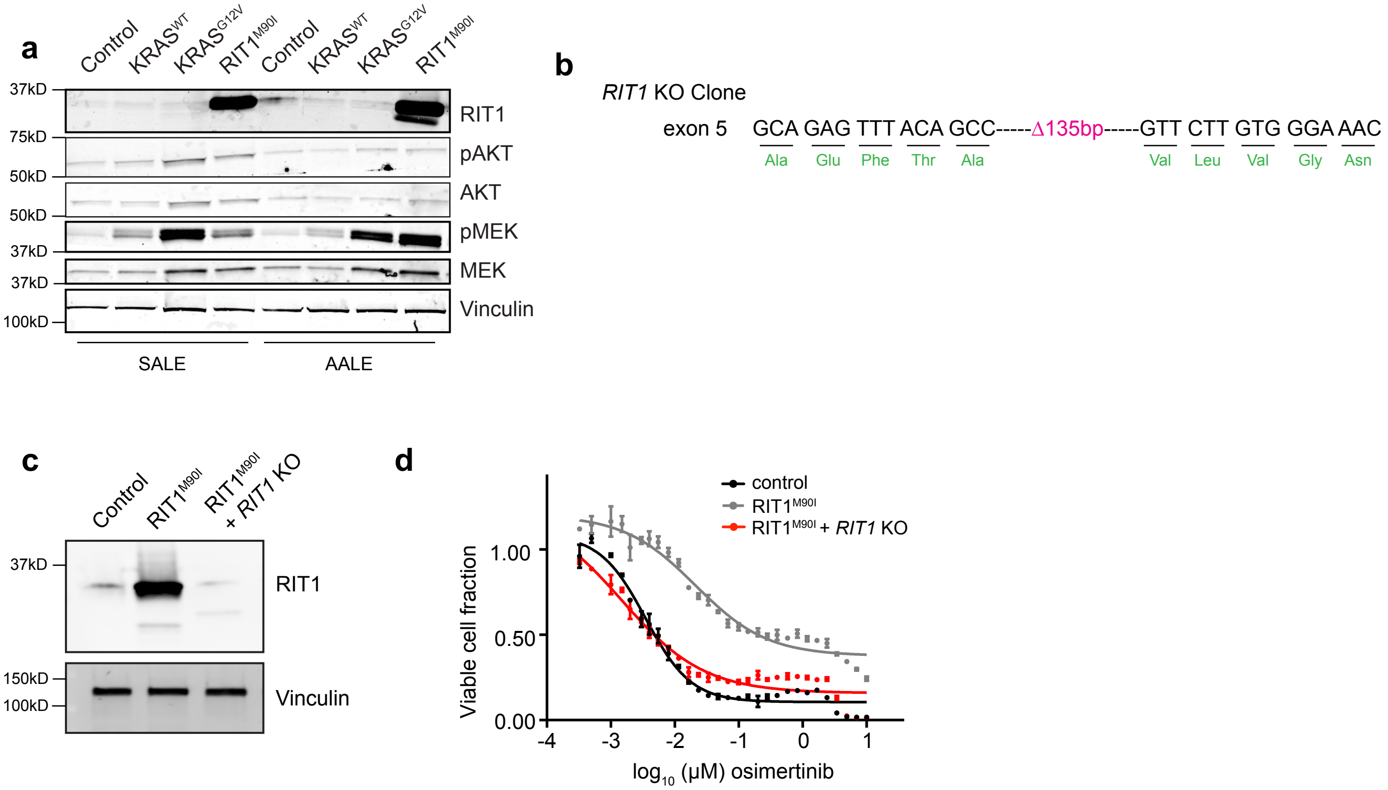

Supplementary Fig. 1. a, Western blot of lysates from SALE and AALE cells stably expressing wild-type or mutant *RIT1* or mutant *RAS* constructs, and empty vector (control). Vinculin was used as a sample processing control. pAKT, phosphorylated Ser473. p-MEK, phosphorylated Ser217/221. Blot representative of n=3 independent experiments. **b,** Schematic of the 135bp deletion identified by Sanger sequencing and ICE analysis in *RIT1* knockout clones generated at the *RIT1* locus by transfection with three synthetic sgRNAs. **c,** Western blot of RIT1 expression in clonal *RIT1* knockout cells compared to parental PC9-Cas9-RIT1^{M90I} cells or control PC9-Cas9-luciferase cells. Blot representative of n=3 independent experiments. **d,** 96 hour dose-response curve of clonal *RIT1* knockout in osimertinib. Viable cell fraction was determined by CellTiterGlo luminescence of treated vs. DMSO-treated cells. Data shown is the mean $\pm$ s.d. of two technical replicates. Uncropped gels are available as source data.

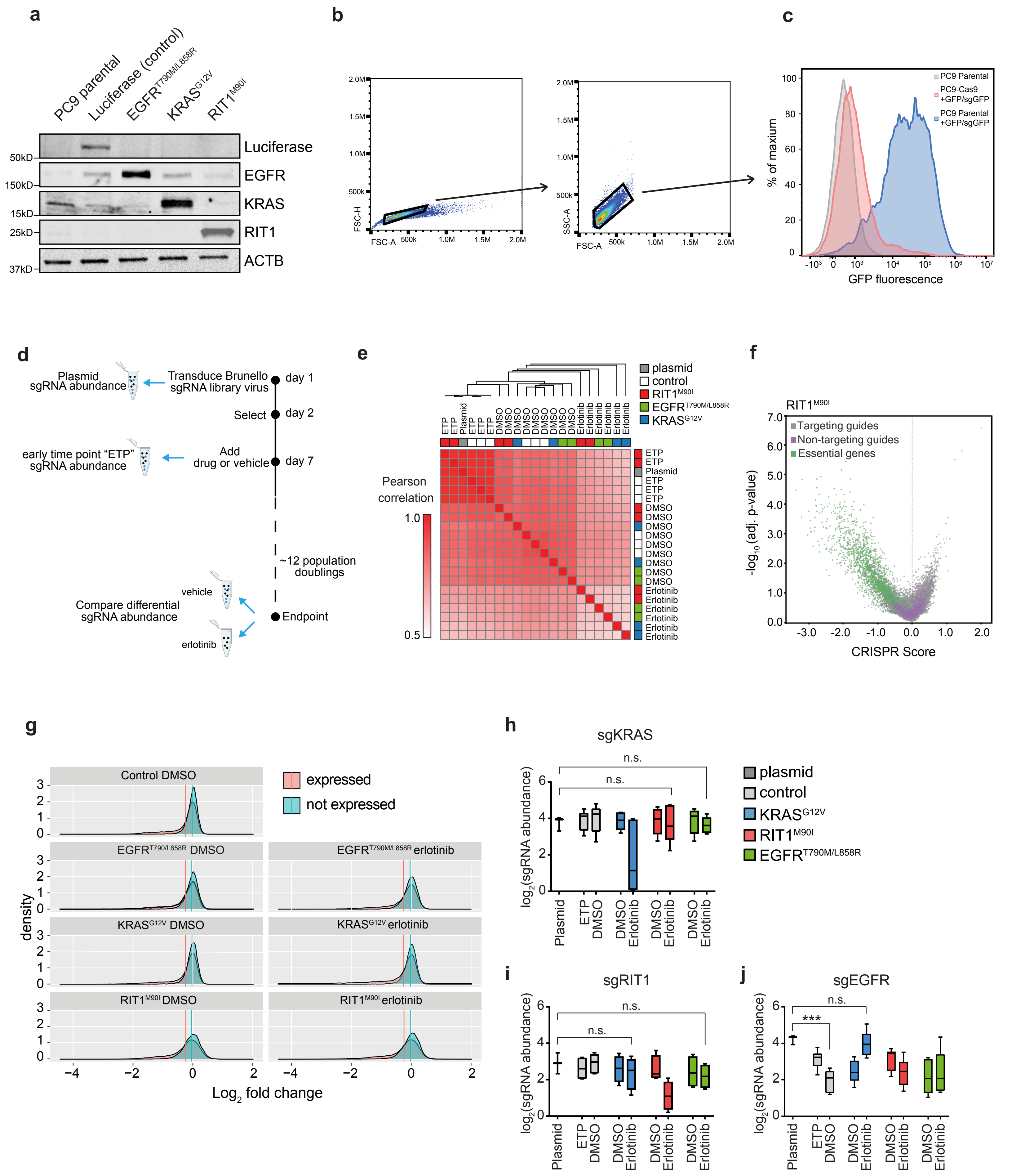
a
PC9 parental
Luciferase (control)
EGFR T790M/L858R
KRAS G12V
RIT1 M90I
Luciferase
EGFR
KRAS
RIT1
ACTB
50kD
150kD
15kD
25kD
37kD
b
FSC-H
FSC-A
SSC-A
c
PC9 Parental
PC9-Cas9 +GFP/sgGFP
PC9 Parental +GFP/sgGFP
% of maxium
GFP fluorescence
d
Plasmid sgRNA abundance
Transduce Brunello sgRNA library virus
day 1
Select
day 2
early time point "ETP" sgRNA abundance
Add drug or vehicle
day 7
~12 population doublings
vehicle
Compare differential sgRNA abundance
Endpoint
erlotinib
e
plasmid
control
RIT1 M90I
EGFR T790M/L858R
KRAS G12V
Pearson correlation
f
RIT1 M90I
Targeting guides
Non-targeting guides
Essential genes
-log10 (adj. p-value)
CRISPR Score
g
Control DMSO
EGFR T790/L858R DMSO
EGFR T790M/L858R erlotinib
KRAS G12V DMSO
KRAS G12V erlotinib
RIT1 M90I DMSO
RIT1 M90I erlotinib
expressed
not expressed
density
Log2 fold change
h
sgKRAS
n.s.
log2(sgRNA abundance)
Plasmid
ETP
DMSO
Erlotinib
plasmid
control
KRAS G12V
RIT1 M90I
EGFR T790M/L858R
i
sgRIT1
j
sgEGFR
***

Supplementary Fig. 2. a, Western blot of lysates generated from parental PC9 cells or stable PC9 cell pools expressing Cas9 and indicated oncogenes. Actin served as a sample processing control. The primary antibodies used are indicated on the right. Blot representative of n=2 technical experiments. **b,** Gating strategy and **c,** distribution of fluorescence in stable Cas9-expressing PC9 cells transduced by EGFP-targeting sgRNAs (red), or non-Cas9 expressing PC9 cells transduced by EGFP-targeting sgRNAs (blue), and non-fluorescent PC9 cells (gray). **d,** Schematic illustrates the workflow of genome-wide CRISPR/Cas9 knockout library screening. On day 1 the human genome-wide Brunello CRISPR/Cas9 knockout library (Brunello) was transduced into isogenic PC9 cells at low MOI. The transduced cells were selected by puromycin on day 2. Cells were cultured in vehicle (DMSO) or erlotinib for ~12 population doublings. Genomic DNA was extracted from erlotinib and vehicle treated cells and the sgRNA sequence was amplified by PCR. Abundance of sgRNAs was determined by Illumina sequencing and analyzed by MAGeCK[35]. Schematic created with BioRender.com. **e,** Pairwise Pearson correlation matrix of sgRNA abundance across screen replicates. Each column/row corresponds to a different replicate. **f,** Volcano plot showing the distribution of targeting guides, essential guides, and control non-targeting guides (collapsed at gene level and/or average of four sgRNA per gene) after normalization. CRISPR Score was calculated as described in the Method. Data shown is from PC9-Cas9-RIT1^{M90I} but similar results were obtained from each cell line. **g,** $\log_2$ fold-change distribution of all sgRNAs targeting expressed genes (red) or non-expressed genes (blue) in each cell line. Vertical lines indicate the mean of each distribution. **h-j,** Expanded view of data shown in Fig. 2d. Box plot showing the $\log_2$sgRNA abundance (reads per million) from sgRNAs targeting **h,** *KRAS*, **i,** *RIT1* or **j,** *EGFR* in isogenic PC9-Cas9 cells by screening condition. Plasmid, starting plasmid library. Control, Firefly luciferase vector control cells. ETP, early time point. DMSO, vehicle control. Box plots show the median (center line), first and third quartiles (box edges), and the min and max range (whiskers) of replicates. *** p = 0.001, calculated by one-sided permutation testing using MAGeCK. For control PC9 cells ETP and DMSO n=3 biological replicates. For oncogene-expressing PC9 cells DMSO and erlotinib n=2 biological replicates. Uncropped gels are available as source data.

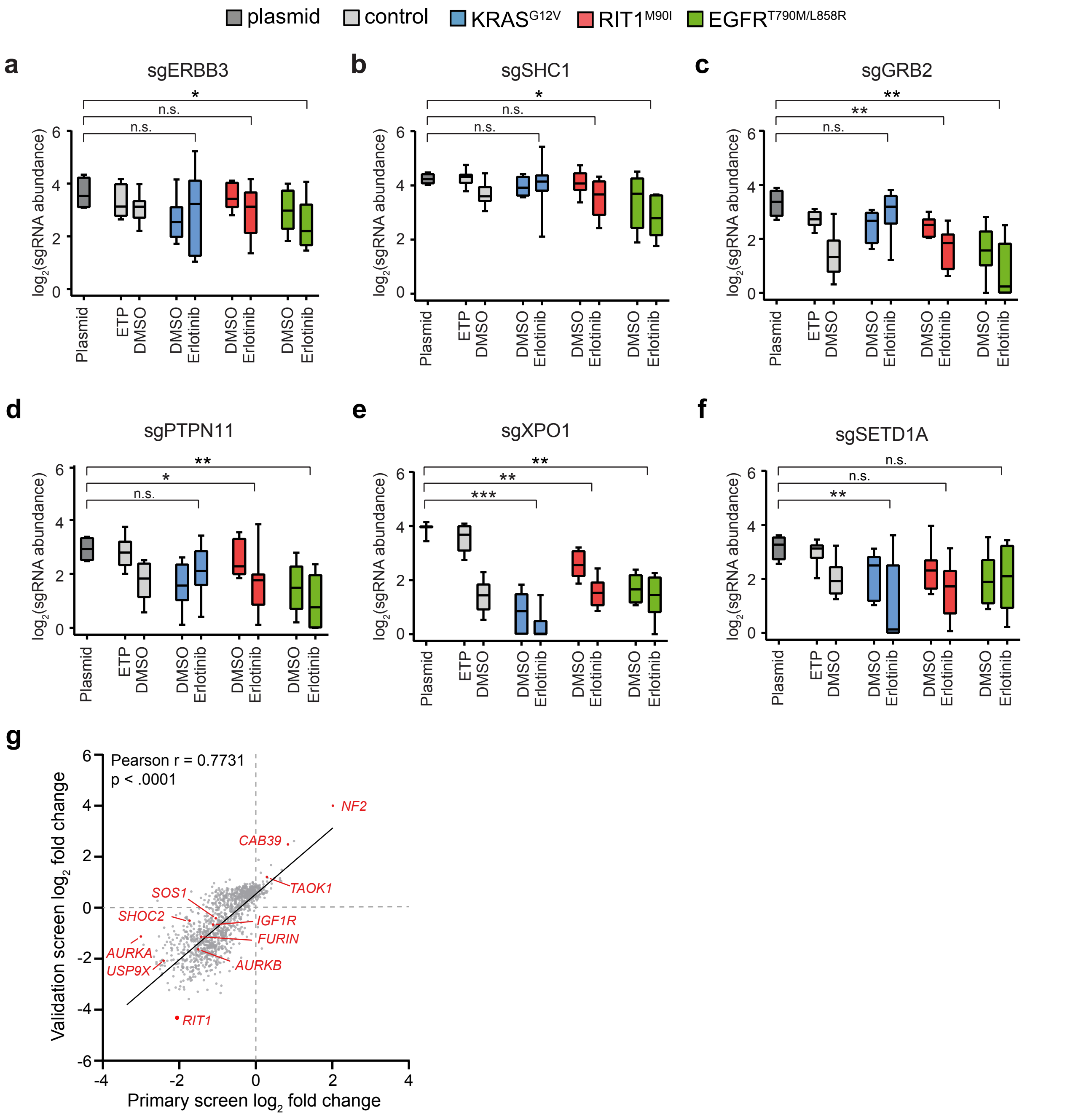

Supplementary Fig. 3. a-f, Box plot showing the $\log_2$sgRNA abundance (reads per million) from sgRNAs targeting key genes discussed in the text. Labeling is as in Supplementary Fig. 2h-j. * p < 0.05, ** p < 0.01, *** p < 0.001 (**a,** * p = 0.037. **b,** * p = 0.033. **c,** ** p = 0.003 (plasmid v. RIT1^{M90I}), ** p = 0.002 (plasmid v. EGFR$^{T790M/L858R}$). **d,** * p = 0.035, ** p = 0.002. **e,** *** p = 1.15e^{-5}, ** p = 0.001 (plasmid vs. RIT1^{M90I}), ** p = 0.004 (plasmid vs. EGFR$^{T790M/L858R}$). **f,** ** p = 0.003) calculated by one-sided permutation testing using MAGeCK. For control PC9 cells ETP and DMSO n=3 biological replicates. For oncogene-expressing PC9 cells DMSO and erlotinib n=2 biological replicates. **g,** Primary and secondary CRISPR screen correlation analysis in PC9-Cas9-RIT1^{M90I} erlotinib-treated cells. The solid black diagonal line displays the linear regression. Relevant RIT1^{M90I} dependencies and cooperating genes discussed in the text are labeled in red.

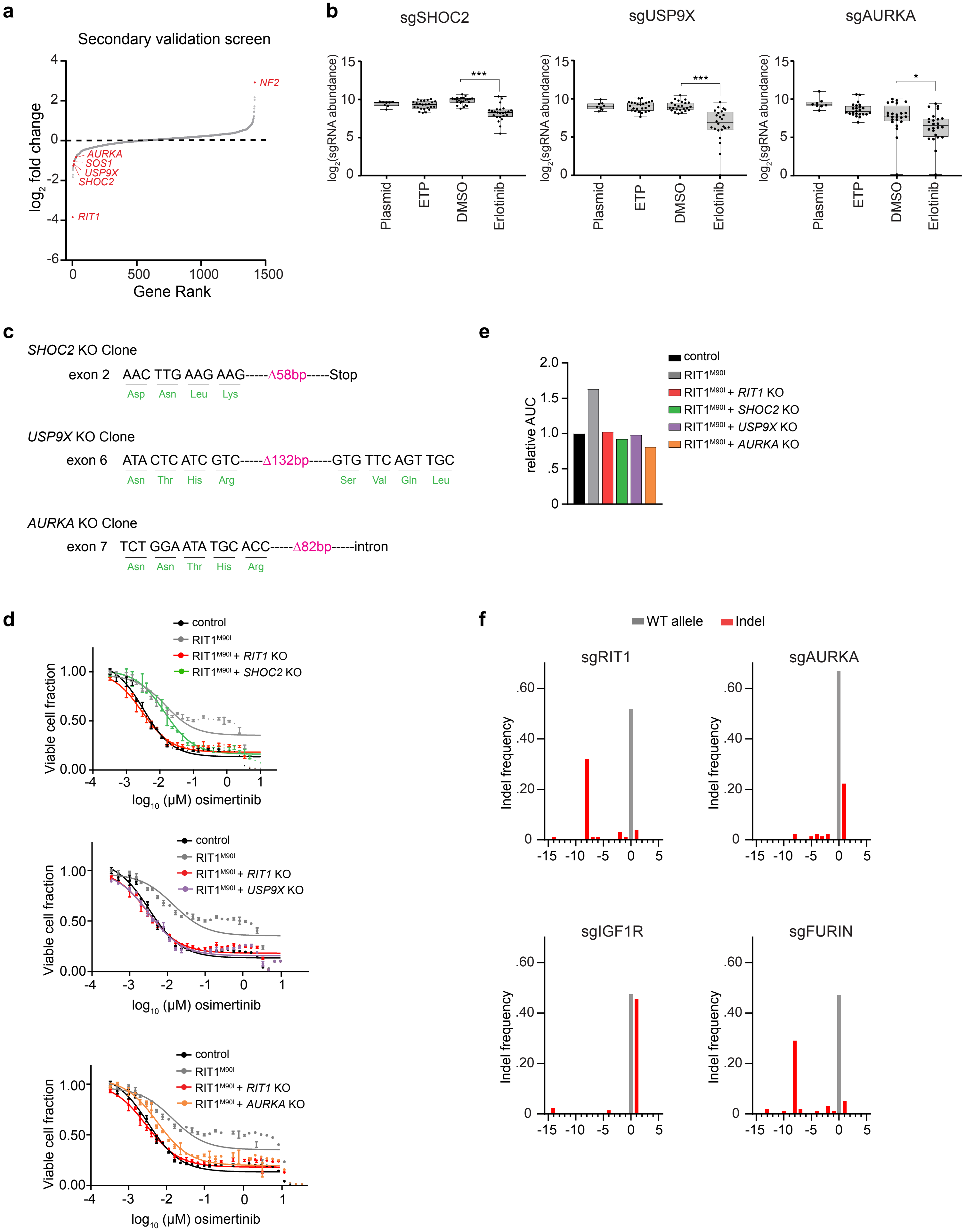
a
Secondary validation screen
log2 fold change
Gene Rank
NF2
AURKA
SOS1
USP9X
SHOC2
RIT1
b
sgSHOC2
sgUSP9X
sgAURKA
log2(sgRNA abundance)
Plasmid
ETP
DMSO
Erlotinib
***
***
*
c
SHOC2 KO Clone
exon 2 AAC TTG AAG AAG-----Δ58bp-----Stop
Asp Asn Leu Lys
USP9X KO Clone
exon 6 ATA CTC ATC GTC-----Δ132bp-----GTG TTC AGT TGC
Asn Thr His Arg Ser Val Gln Leu
AURKA KO Clone
exon 7 TCT GGA ATA TGC ACC-----Δ82bp-----intron
Asn Asn Thr His Arg
d
control
RIT1M90I
RIT1M90I + RIT1 KO
RIT1M90I + SHOC2 KO
RIT1M90I + USP9X KO
RIT1M90I + AURKA KO
Viable cell fraction
log10 (μM) osimertinib
e
relative AUC
control
RIT1M90I
RIT1M90I + RIT1 KO
RIT1M90I + SHOC2 KO
RIT1M90I + USP9X KO
RIT1M90I + AURKA KO
f
WT allele
Indel
sgRIT1
sgAURKA
sgIGF1R
sgFURIN
Indel frequency

Supplementary Fig. 4. a, Rank plot of the mean difference in CRISPR score of PC9-Cas9-RIT1^{M90I} erlotinib-treated and vehicle (DMSO) treated cells of secondary validation screen. Selected essential genes are labeled in red. **b,** Validation of *SHOC2*, *USP9X*, and *AURKA*, dependencies in PC9-Cas9-RIT1^{M90I}. Data shown is 8 sgRNAs across three biological replicates of each condition. * $p < 0.05$, *** $p < 0.001$ (*** $p = 0.0002$ (sgSHOC2), *** $p = 0.0008$ (sgUSP9X), * $p = 0.034$), calculated by unpaired two-tailed t-test. **c,** Schematic of the deletions identified by Sanger-sequencing and ICE analysis in *SHOC2*, *USP9X*, and *AURKA* knockout clones generated by transfection with synthetic multi-guide sgRNA guides in PC9-Cas9-RIT1^{M90I} cells. **d,** 96-hour dose-response curve of osimertinib in clonal *SHOC2* knockout (KO), *USP9X* KO, or *AURKA* KO cells derived from PC9-Cas9-RIT1^{M90I} cells. The same data for control and PC9-Cas9-RIT1^{M90I} cells is plotted on each panel for reference. Data shown is the mean ± s.d. of 2 technical replicates. **e,** Area under the curve (AUC) analysis of data from panel (e). **f**, Histogram representing the frequency of indel lengths induced by Cas9 in NCI-H2110 expressing stated sgRNA after 5 days of doxycycline treatment.

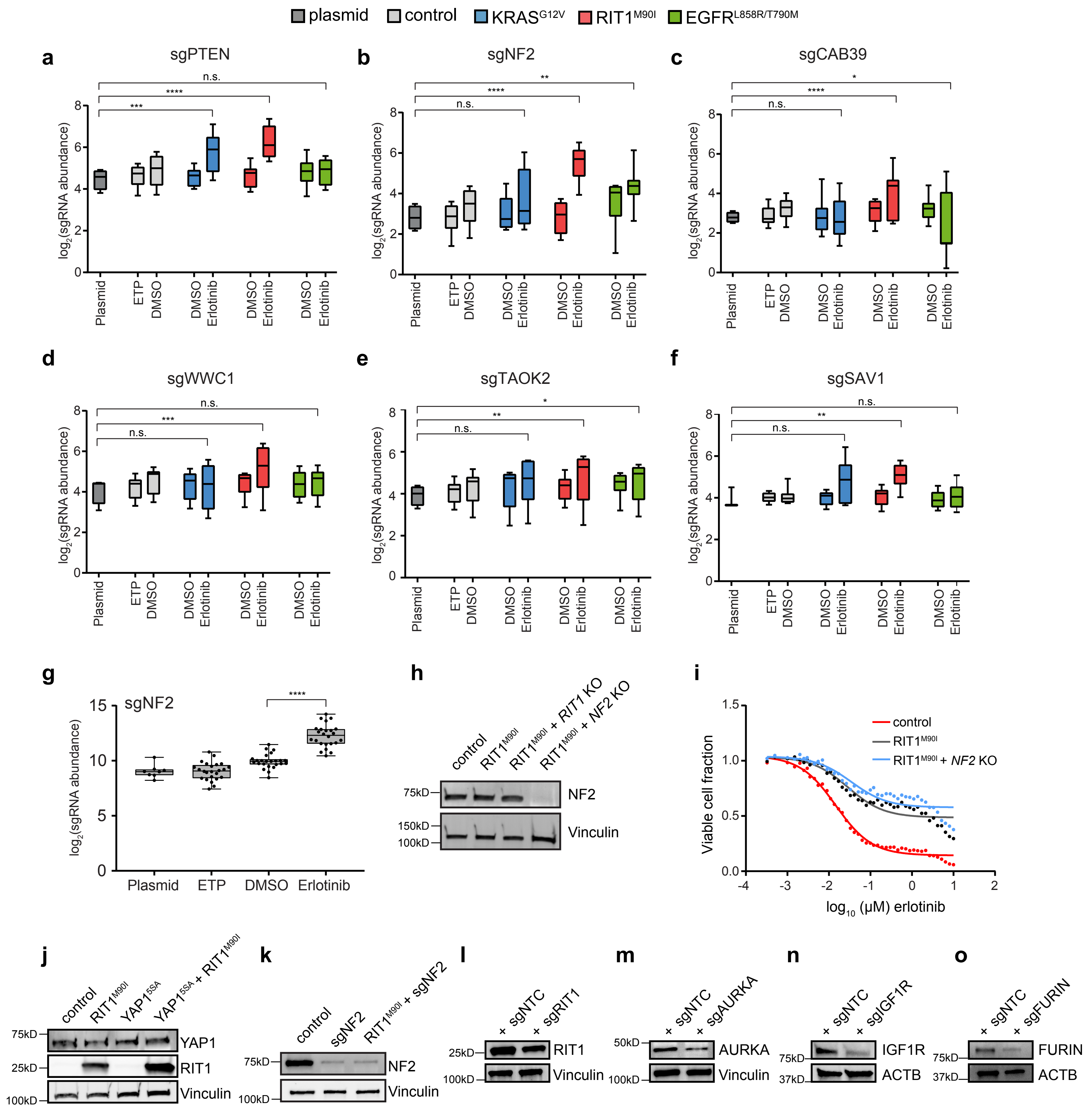

plasmid
control
KRAS G12V
RIT1 M90I
EGFR L858R/T790M
a
sgPTEN
b
sgNF2
c
sgCAB39
d
sgWWC1
e
sgTAOK2
f
sgSAV1
log2(sgRNA abundance)
Plasmid
ETP
DMSO
Erlotinib
n.s.
g
sgNF2
h
control
RIT1 M90I
RIT1 M90I + RIT1 KO
RIT1 M90I + NF2 KO
NF2
Vinculin
i
control
RIT1 M90I
RIT1 M90I + NF2 KO
Viable cell fraction
log10 (μM) erlotinib
j
YAP1 5SA
YAP1 5SA + RIT1 M90I
YAP1
RIT1
k
sgNF2
RIT1 M90I + sgNF2
l
+ sgNTC
+ sgRIT1
m
+ sgAURKA
AURKA
n
+ sgIGF1R
IGF1R
ACTB
o
+ sgFURIN
FURIN

Supplementary Fig. 5. a-f, Box plot showing the $\log_2$sgRNA abundance (reads per million) from sgRNAs targeting key Hippo pathway genes across isogenic PC9-Cas9 cells line by condition. Labeling is as in Supplementary Fig. 2h-j. * $p < 0.05$, ** $p < 0.01$, *** $p < 0.001$, **** $p < 0.0001$ (**a,** *** $p = 0.0009$, **** $p = 0.000002$. **b,** **** $p = 0.0000002$, ** $p = 0.0042$. **c,** **** $p = 0.000003$, * $p = 0.04$. **d,** *** $p = 0.0004$. **e,** ** $p = 0.001$, * $p = 0.0239$. **f,** ** $p = 0.002$), calculated by one-sided permutation testing using MAGeCK. **g,** Validation of *NF2* positive selection from the secondary validation screen in PC9-Cas9-RIT1^{M90I} cells. Data shown is 8 sgRNAs across three biological replicates of each condition. Box plots show the median (center line), first and third quartiles (box edges), and the min and max range (whiskers) of replicates, **** $p = 0.0000003$ by unpaired two-tailed t-test. **h,** Western blot of lysates from control, PC9-Cas9-RIT1^{M90I}, PC9-Cas9-RIT1^{M90I} + *RIT1* KO, and PC9-Cas9-RIT1^{M90I} + *NF2* KO cells. Vinculin was used as a loading control. **i.** Dose-response curve of erlotinib in PC9-Cas9-RIT1^{M90I} + *NF2* knockout cell pools. Cells were treated with erlotinib for 96-hours. Viable cell fraction was determined by CellTiterGlo luminescence of treated cells normalized to the average value of DMSO-treated cells. Data shown is the mean + s.d. of 2 technical replicates. **j,** Western blot of lysates from SALE cells used for the xenograft assay in Fig. 7g. Vinculin was used as a loading control. **k,** Western blot of lysates from SALE cells used for the xenograft assay in Fig. 7h. Vinculin was used as a loading control. **l-o**, Western blot of lysates from RIT1^{M90I} + YAP1^{5SA} cells transduced with control non-targeting (NTC) or the indicated gene-specific sgRNA and induced with doxycycline in vitro for 5 days. Vinculin or beta-Actin (ACTB) was used as a loading control. Uncropped gels are available as source data.

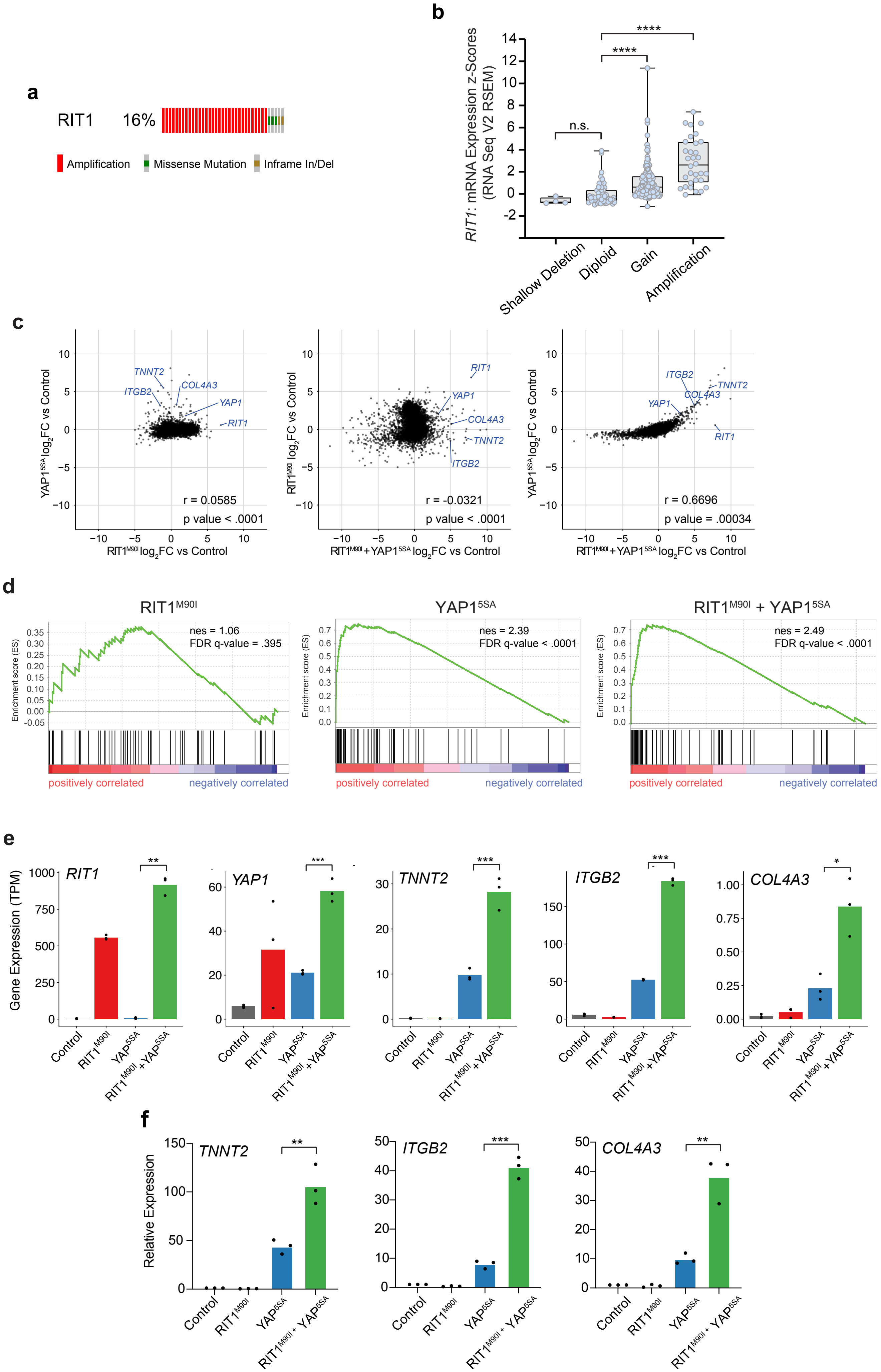
a
RIT1 16%
Amplification
Missense Mutation
Inframe In/Del
b
RIT1: mRNA Expression z-Scores (RNA Seq V2 RSEM)
n.s.
****
****
Shallow Deletion
Diploid
Gain
Amplification
c
YAP1^5SA log2FC vs Control
RIT1^M90I log2FC vs Control
r = 0.0585
p value < .0001
TNNT2
COL4A3
ITGB2
YAP1
RIT1
RIT1^M90I log2FC vs Control
RIT1^M90I + YAP1^5SA log2FC vs Control
r = -0.0321
p value < .0001
YAP1^5SA log2FC vs Control
RIT1^M90I + YAP1^5SA log2FC vs Control
r = 0.6696
p value = .00034
d
RIT1^M90I
nes = 1.06
FDR q-value = .395
YAP1^5SA
nes = 2.39
FDR q-value < .0001
RIT1^M90I + YAP1^5SA
nes = 2.49
FDR q-value < .0001
Enrichment score (ES)
positively correlated
negatively correlated
e
Gene Expression (TPM)
RIT1
YAP1
TNNT2
ITGB2
COL4A3
Control
RIT1^M90I
YAP^5SA
RIT1^M90I +YAP^5SA
f
Relative Expression
TNNT2
ITGB2
COL4A3
Control
RIT1^M90I
YAP^5SA
RIT1^M90I + YAP^5SA

Supplementary Fig. 6. a, Frequency of *RIT1* amplification and mutation (n=37) from the TCGA lung adenocarcinoma paper[6] , visualized using cBioPortal (http://cbio.mskcc.org). Each column represents a sample. Samples lacking alterations (n=193) in *RIT1* are not shown. 16% indicates the percentage of samples in the cohort with mutation or amplification in *RIT1* (37/230). **b,** Box plot showing the relationship between *RIT1* mRNA abundance and copy number of *RIT1* from TCGA analysis shown in (j). Diploid, two alleles present; Gain, low-level gene amplification event; Amplification, high-level gene amplification event. **** $p < 0.0001$ (diploid vs. gain: **** $p = 7.3338e^{-6}$, diploid vs. amplification: **** $p = 2.9354e^{-13}$ by unpaired two-tailed t-test). **c,** Pairwise comparisons of transcriptomic changes due to RIT1^{M90I}, YAP1^{5SA}, or RIT1^{M90I} + YAP1^{5SA} compared to control. Differential expression is plotted as $\log_2$(fold-change). Pearson correlations are shown. For RIT1^{M90I} compared to YAP^{5SA} $p = 6.155x10^{-11}$, for RIT1^{M90I} compared to RIT1^{M90I} +YAP^{5SA} $p < 2.2x10^{-16}$. **d,** Gene Set Enrichment Analysis of the Cordenosi YAP signature in differentially expressed genes in RIT1^{M90I}, YAP1^{5SA}, or RIT1^{M90I} + YAP1^{5SA} compared to control. **e,** Transcript levels from RNA-seq of *RIT1*, *YAP1*, and *YAP1*-regulated genes *TNNT2*, *ITGB2*, *COL4A3*. Data shown is the average transcripts per million (TPM) three individ-ual replicates (points). n.s., $p > 0.05$, * $p < 0.05$, ** $p<0.01$, *** $p < 0.001$, by unpaired two-tailed t-test. For *RIT1*, ** $p = 0.0044$; for *YAP1*, *** $p = 0.00049$; for *TNNT2*, *** $p = 0.00061$; for *ITGB2*, *** $p = 4.16x10^{-6}$ for *COL4A3*, * $p = 0.0139$, by unpaired two-tailed t-test. **f,** Quantitative RT-PCR validation of *TNNT2*, *ITGB2*, and *COL4A3* relative expression in SALE cell lines analyzed in (e). Data shown is the average of three biological replicates (points) after normalization to *18S* expression. For *TNNT2*, ** $p = 0.0078$; for *ITGB2*, *** $p = 0.0001$; for *COL4A3*, ** $p = 0.0038$ by unpaired two-tailed t-test.

Supplementary Table 1. Sequence of each multi-guide sgRNA within the multiplex library from Synthego.

**guide_label**	**guide_sequence**	**guide_number**	**gene**	**seqid**	**cutsite**	**transcript_id**
AURKA+56381415	TAAGTGTTCATTTATTGTCC	1	AURKA	chr20	56381415	ENST00000312783.10
AURKA+56381497	ATTCTGGAATATGCACCACT	2	AURKA	chr20	56381497	ENST00000312783.10
AURKA+56381551	CCTAATATTCTTAGACTGTA	3	AURKA	chr20	56381551	ENST00000312783.10
NF2-29661210	TTGGATCCACAGAATAAAAA	1	NF2	chr22	29661210	ENST00000338641.8
NF2+29661249	GGTCATAAATGTGAAGCCCC	2	NF2	chr22	29661249	ENST00000338641.8
NF2+29661303	TGTTTCGGATTTCATTCCAC	3	NF2	chr22	29661303	ENST00000338641.8
RIT1-155904353	TTGTTTCCCACAAGAACCAC	1	RIT1	chr1	155904353	ENST00000368323.7
RIT1-155904411	GTTTAAACTCACGAACTTCA	2	RIT1	chr1	155904411	ENST00000368323.7
RIT1+155904488	AGGCAGAGTTTACAGCCATG	3	RIT1	chr1	155904488	ENST00000368323.7
SHOC2-110964869	TTGATAACTTGAAGAAGCTG	1	SHOC2	chr10	110964869	ENST00000369452.8
SHOC2-110964927	TCCTTCAGTGGTGTATAGGC	2	SHOC2	chr10	110964927	ENST00000369452.8
SHOC2-110964981	TCGTATAACTACTGTGGAAA	3	SHOC2	chr10	110964981	ENST00000369452.8
USP9X-41136832	TAACAATACTCATCGTCTGG	1	USP9X	chrX	41136832	ENST00000378308.6
USP9X+41136904	GAATTTGCAATGAGGATTTA	2	USP9X	chrX	41136904	ENST00000378308.6
USP9X+41136964	TTCAGGCAACTGAACACTTG	3	USP9X	chrX	41136964	ENST00000378308.6

Supplementary Table 2. Sequence of each sgRNA used to generate individual gene knockouts in H2110iCas9 and SALEiCas9 cells.

**gene_target**	**guide_sequence**
NTC (non-targeting control)	AAAATAGCAGTAAACTCAAC
RIT1	CAGGTGTATCGTCAGTACGT
NF2	GCTTGGTACGCAGAGCACCG
AURKA	CCATATAGAAAATAATCCTG
IGF1R	GGAGAACGACCATATCCGTG
FURIN	CAACGTGCCGTGGTACAGCG

Supplementary Table 3.

Primer Name	Sequence
P5	AATGATACGGCGACCACCGAGATCTACACTCTTTCCCTACACGACGCTC TTCCGATCT[s]TTGTGGAAAGGACGAAACACCG
P7	CAAGCAGAAGACGGCATACGAGAT[NNNNNNNN]GTGACTGGAGTTCAGAC GTGTGCTCTTCCGATCTTCTACTATTCTTTCCCCTGCACTGT

P5/P7 primers were purchased from Integrated DNA Technologies (IDT).
[s] = stagger region, [NNNNNNNN] = barcode.

Supplementary Table 4. Primer sequences used for Sanger sequencing of pooled and clonal individual gene knockouts in PC9-Cas9 cells.

Gene	Forward Oligo	Reverse Oligo
AURKA	CAGATTCTAAAGTGGGGTTTGG	CCTGGGCAACATAACAAGACC
NF2	GCCTGCTTATTTAACCAGAGGG	CAACAACCACACCCTCAAAGCC
RIT1	CGTATTGATGATGAGCCTGC	CACAAAGGGAGGGAAAGAGA
SHOC2	CAGCAGAGGTGGGATGTTTA	CCAAGGCCTCTTACCAATTTCAGC
USP9X	GGCGTCAGACTTCTGCATTT	GCTTCTCCTTGATTCGCAAC

Supplementary Table 5. Primer sequences used for Sanger sequencing of pooled gene knockouts in H2110iCas9 and SALEiCas9 cells.

**Gene**	**Forward Oligo**	**Reverse Oligo**
RIT1	CGTATTGATGATGAGCCTGC	CACAAAGGGAGGGAAAGAGA
NF2	TGAGCTGTTTGCAGTGGCGAGG	CGCCACTGCATTTCAGCCTGGA
AURKA	ACTCTCACACCTGACGGCAGCT	CCACCAGCCGTAGTCAACCTGC
FURIN	TCCTGGCCTCAAGCGATCCTCC	ATTGGGGCCTGTGATGGGTGGA
IGF1R	GTGTGACATGCTGGGCCTCTGG	AACAAGGGCAGCTGAAGACGGC
