# Supplementary material for: Integrative oncogene-dependency mapping identifies RIT1 vulnerabilities and synergies in lung cancer
Source: Nat Commun. 2021 Aug 9;12:4789. doi: 10.1038/s41467-021-24841-y (PMC8352964; doi:10.1038/s41467-021-24841-y)
Supplement: Supplementary file 2 — Description of Additional Supplementary Files [file 41467_2021_24841_MOESM2_ESM.pdf]

Description of Additional Supplementary Files

Title: Supplementary Data 1. Sequencing results from CRISPR screens performed in isogenic PC9 cells.

Description: The 'Log2RPM' tab provides the sgRNA barcode sequences included in the Brunello library and the abundance of each sgRNA in the plasmid library and at the conclusion of each screen in $\log_2$(RPM) where RPM = reads per million. The 'SSMD' tab provides the median strictly standardized mean difference (SSMD) statistic for each screen arm. ETP = early time point, ERLO = erlotinib

Title: Supplementary Data 2. Normalized CRISPR score data from CRISPR screens performed in isogenic PC9 cells.

Description: The 'CRISPR score' tab provides the averaged and replicate CRISPR score (a normalized value relating abundance of gene-targeting sgRNAs), and $-\log_{10}$ p-values at the gene-level for each screen. The 'Normalization' column refers to the list of essential and non-essential genes obtained from DepMap used for normalization (Release DepMap Public 20Q2, see methods). Essential genes were defined using this matrix with a threshold of $CS<-0.5$ and $p < 0.05$. CS = CRISPR Score, ERLO = erlotinib

Title: Supplementary Data 3. Table of baseline synthetic lethal genes identified from CRISPR screens performed in isogenic PC9 cells.

Description: List of baseline synthetic lethal genes in each cellular context showing essential genes in oncogene-expressing cells ($CS < -0.5$ and $p < 0.05$) that were not essential in the control DMSO-treated PC9-luciferase cells. Note that "baseline synthetic lethal" refers to new dependencies conferred by each oncogene mutation in "baseline" control DMSO-treated cells.

Title: Supplementary Data 4. Table of essential genes associated with Venn diagram in Figure 3A.

Description: List of essential genes ($CS < -0.5$ and $p < 0.05$) for each screen comparing plasmid library to erlotinib-treated cells.

Title: Supplementary Data 5. Analysis of targeted validation screen carried out in PC9-Cas9 $RIT1^{M90I}$.

Description: The 'Averaged validation screen data' tab provides the provides the averaged LFC, FDR, p-value, and $-\log_{10}$ p-values at the gene-level for validation screen at Early Time Point (ETP), DMSO-, and erlotinib-treated PC9-Cas9 $RIT1^{M90I}$ cells. The 'sgRNA barcodes' tab provides the sgRNA barcode sequences of the secondary validation library. The id 'RIT1nocDNA' refers to the sgRNAs in the library that don't target the RIT1 cDNA transgene.

Title: Supplementary Data 6. Small molecule library information pertaining to drug screen performed in isogenic PC9 cells.

Description: The 'AUC' tab provides the area-under-the-curve data presented in Fig. 5c and corresponding statistical analysis for PC9-RIT1^{M90I} and PC9-KRAS^{G12V} cells treated with 500 nM erlotinib in combination with each indicated agent. The 'Compound info' tab provides name, clinical investigation status, target, and molecular structure of each compound in library. The 'Compound Plate Map' tab provides the layout of each plate for the small molecule library The 'Renilla_norm', 'RIT1M90I_norm', and 'KRASG12V_norm' tabs provides data from each condition and replicate from the drug screen normalized by dividing each well's CellTiterGlo luminescence with the average CellTiterGlo luminescence of a plate treated only with 500 nM erlotinib.

Title: Supplementary Data 7. KEGG pathway analysis of significantly under-expressed mRNAs in *RIT1*-mutant and *RIT1*-amplified lung adenocarcinomas from TCGA.

Description: The 'KEGG pathway analysis' tab list pathways (FDR < .05) that were enriched among the 1015 genes under-expressed in *RIT1*-altered tumors (q<=0.25).

Title: Supplementary Data 8. MSK-IMPACT clinical cohort information.

Description: Table provides demographic, somatic alteration information, and H-scores for *RIT1*-mutant, *YAP1/YES1*-amplified, and control non-small cell lung tumor samples analyzed in Figure 8.

Title: Supplementary Data 9. Differential expression from RNA sequencing of SALE-RIT1^{M90I}, SALE-YAP1^{5SA}, and SALE-RIT1^{M90I}+YAP1^{5SA}.

Description: Provides average $\log_2$(fold change) and p value for all 12,458 genes analyzed in three replicates of SALE-RIT1^{M90I}, SALE-YAP1^{5SA}, and SALE-RIT1^{M90I}+YAP1^{5SA} compared to parental control.

Title: Supplementary Data 10. DepMap gene lists of common essential and nonessential genes.

Description: Provides gene lists (common essentials and nonessentials) from DepMap.org.
